# Developing a partner selection framework for digital agricultural science and technology innovation alliances

**DOI:** 10.1038/s41598-026-47641-0

**Published:** 2026-04-08

**Authors:** Mingqiu Li, Jinqiu Li, Yuyou Zou

**Affiliations:** 1 College of Economics and Management, Northeast Forestry University, Harbin, 150040 China; 2 School of Economics and Management, Harbin Engineering University, Harbin, 150001 China

**Keywords:** Digital agricultural science and technology, Innovation alliance, Partner selection, Field theory, Information entropy, Orthogonal projection, Engineering, Mathematics and computing

## Abstract

Selecting appropriate collaborators represents a critical factor in ensuring optimal innovation outcomes for Digital Agricultural Science and Technology (DAST) alliances. The partner identification process for DAST innovation coalitions constitutes a multi-criteria decision analysis (MCDA) challenge characterized by temporal dynamics and information ambiguity. This study proposes a time-weighted orthogonal projection approach within dynamic intuitionistic fuzzy environments to address this challenge. This approach fully considers decision-making rules and resource complementarity: First, it allocates weights by integrating the time dimension with multi-objective optimization model construction, fully absorbing decision information at different stages through time weight solutions to reduce uncertainty in multi-stage information collection; Second, it employs fuzzy set theory and orthogonal projection method to evaluate members and candidate partners. Based on this theoretical foundation, this paper proposes a field model incorporating resource complementarity for selecting optimal partners. This novel field model can assist agricultural science and technology innovation alliances in implementing collaborative innovation practices, while simultaneously providing decision-making guidance for optimizing dynamic partner selection models to build long-term stable partnership relationships. Practical validation through China’s 5G Agricultural Digitalization Alliance case study demonstrates the methodology’s practicality and operational efficacy.

## Introduction

The advancement of information technologies including big data analytics, Internet of Things (IoT) systems, blockchain networks, and 5G connectivity has driven the evolution from conventional farming practices to digitally-enabled agricultural systems^1,2^. This technological transformation serves as a crucial catalyst for fostering cross-sector collaboration, enhancing agricultural productivity^3,4^, and stimulating rural economic growth^5,6^. Data from the 2023 China Digital Economy Development Report reveals significant disparities in industrial digitization, with agricultural digitalization levels reaching only 10.5% in 2022—substantially below average sector benchmarks. This developmental gap stems from inadequate digital infrastructure, insufficient technical expertise, and resource allocation challenges that have hindered the pace of digital integration^1,6^. To address these challenges and promote sustainable agricultural modernization, Chinese policymakers have implemented strategic initiatives including the Digital Rural Development Strategy Framework, the 2019–2025 Digital Agriculture Implementation Plan, and the 2022–2025 Rural Digitalization Action Agenda. The 2023 Central Policy Document emphasizes "accelerating agricultural big data applications and advancing intelligent farming systems", marking a strategic shift from theoretical planning to practical implementation. These coordinated efforts position digital agriculture as both an enabler of eco-friendly agricultural practices and a transformative force in modern agri-tech innovation, demonstrating significant potential for driving technological advancement and comprehensive rural revitalization.

Agricultural Science and Technology (AST) innovation represents an open collaborative framework deeply intertwined with industry-university-research (I-U-R) partnerships in agriculture. This model enables enterprises, academic institutions, research organizations, and independent agricultural innovators to harness and synergize their strengths, thereby expediting the advancement and practical implementation of agricultural technologies^7^. These AST innovation coalitions have emerged as quintessential embodiments of I-U-R collaboration. Globally, national governments have prioritized cooperative AST development as a catalyst for modern agricultural progress. China’s agricultural administration demonstrated this commitment through its 2014 Central Rural Working Conference, which facilitated the establishment of the National Agricultural Science and Technology Innovation Coalition. Subsequently, the 2015 inaugural policy document from Chinese central authorities outlined strategic plans for AST collaborative innovation networks^7^. Multidisciplinary experts have actively driven the implementation of agricultural I-U-R partnerships, particularly emphasizing enhanced cooperation between agribusinesses, agricultural universities, specialized research institutes, and innovation organizations. Under the guidance of prominent agricultural research bodies, China has instituted numerous AST coalitions spanning diverse domains including crop genetic resources, agricultural biotechnology, smart farming equipment, ecological sustainability, food safety protocols, and agricultural data analytics. This evolution has transformed AST innovation from conventional linear models to dynamic, networked collaboration frameworks^8^. Building on this momentum, China’s 2019 ministerial directive initiated third-party evaluations of 62 pre-2018 alliances, culminating in the accreditation of 34 pioneering coalitions. These encompassed industrial, regional, and specialized alliances such as the Cotton Industry Coalition, Arid Zone Ecological Agriculture Coalition, and Agricultural Big Data Consortium. The subsequent 2020 policy guidelines further institutionalized alliance development mechanisms while emphasizing their critical role in AST innovation. Most recently, the 2023 accreditation cycle evaluated coalition organizational effectiveness, recognizing 30 exemplary alliances. This sustained policy focus underscores the global prioritization of deep I-U-R integration, collaborative AST innovation, and effective translation of technological breakthroughs into agricultural productivity enhancements – areas receiving heightened attention from both governmental and academic spheres.

Given this context, what strategies can foster mutual trust and enduring collaborations within digital agricultural science and technology (DAST) innovation partnerships? Can the DAST innovation alliance effectively accelerate and enhance the advancement of agricultural technological innovation capabilities? What criteria should guide the selection of appropriate collaborators to optimize the DAST alliance’s innovative outcomes? Existing scholarly works have yet to address the concept of DAST innovation alliances. This research aims to bridge this knowledge gap by investigating these critical questions. This study’s primary contribution lies in introducing the DAST innovation alliance framework. We develop a partner selection model that examines evolving collaboration dynamics, followed by establishing a systematic mechanism to analyze alliance formation processes. The research further validates the proposed methodology’s effectiveness through comprehensive empirical evaluation. Therefore, taking dynamic intuitionistic fuzzy decision-making and field theory as basis, this paper constructs a dynamic selection model for cooperative partners in DAST innovation alliance. This paper mainly has made the following contributions: to have proposed a comprehensive evaluation index framework for partner selection in DAST innovation alliance and to establish a field model of cooperative innovation capabilities for innovation partner selection by integrating dual empowerment and field theory. Firstly, the fuzzy set theory is applied, and the intuitionistic fuzzy entropy method and grey correlation analysis method are employed to tackle the weight vector of attributes. Secondly, the information entropy theory and the TOPSIS method are utilized to address the weight vector of time. By applying the orthogonal projection and replacing the Euclidean distance with the vertical distance, the ecological niche of candidates is evaluated. On this basis, a cooperative innovation capability field model incorporating resource complementarity and dynamic decision-making is proposed to select the best partners. Case studies demonstrate that the cooperative innovation capability field model can be put to use in of innovation partners in the DAST innovation alliance and help produce more realistic selection results. In addition, this paper broads the application of field theory that considers fuzzy decision information and resource complementarity in the collaborative innovation paradigm.

## Materials and methods

### Literature review

#### Digital agriculture innovation management

Digital agriculture involves the comprehensive digitization of agricultural value chains, leveraging computational and communication technologies to equip farmers with enhanced information access, innovative services, and improved opportunities for boosting agricultural profitability and ecological sustainability^9^. First conceptualized in 1997, this paradigm represents technology-intensive farming methodologies augmented by geospatial systems and advanced information infrastructures^10^. This approach integrates digital tools and resources to optimize operational efficiency, enhance economic viability for agricultural practitioners, and strengthen market competitiveness of farm products through technological integration^7^. Academic consensus regarding digital agriculture’s conceptual boundaries remains elusive, and some scholarly attention has expanded to encompass digital infrastructure development^11^, agricultural digitization processes^12,13^, and digital industrialization strategies^14^. This growing academic engagement signals digital transformation’s critical role in advancing agriculture toward high-quality economic development.

The concept of AST innovation alliances lacks a universally accepted definition, with DAST innovation alliances representing an even more novel construct. Functioning as a subset of industrial innovation alliances, these entities also embody strategic collaborative frameworks. The foundational notion of strategic alliances originated from Hopland (DEC Corporation’s former CEO) and management scholar Nigel, who characterized them as collaborative entities formed by multiple enterprises sharing strategic objectives and comparable operational capacities. Subsequent researchers refined this concept, describing strategic alliances as flexible cooperative arrangements where members leverage complementary strengths and risk-sharing mechanisms through contractual agreements to achieve shared objectives like market expansion and resource optimization^15^. Contemporary research on AST innovation primarily examines four dimensions: innovation imperatives, methodological approaches, operational mechanisms, and strategic implementations. Pioneering work by researchers introduced the induced technological innovation theory, positioning technological advancement as the central driver of agricultural progress^16^. Subsequent studies have reinforced this perspective, with scholars emphasizing agricultural technological innovation’s critical role in sustaining industry development and rural economic growth^17,18^. Recent investigations highlighted collaborative partnerships between agricultural producers and Non-Governmental Organizations (NGOs) have been shown to enhance specialized technology development and knowledge-sharing networks among stakeholders^19^. Emerging research directions include optimizing resource allocation strategies for green agricultural innovation^20^ and analyzing diffusion mechanisms of agricultural technological advancements through specialized innovation hubs like agricultural science parks^21^. These diverse scholarly efforts collectively contribute to our understanding of innovation dynamics in agricultural systems, addressing both theoretical frameworks and practical implementation challenges. We proposed articulate three specific dimensions that distinguish DAST alliances from conventional strategic alliances, R&D consortia, and I-U-R collaborations: (a) Data-centric governance logic. Unlike traditional alliances where resource complementarity involves bargaining over scarce assets^22^, DAST alliances operate on data non-rivalry—agricultural data can be simultaneously used by multiple parties without depreciation, creating unique governance challenges around data sovereignty, privacy, and value attribution^23^. (b) Algorithmic co-production. While R&D consortia typically separate research and application phases, DAST alliances feature continuous algorithmic iteration where machine learning models are trained, validated, and refined through real-time agricultural data streams, constituting a “living laboratory” innovation mode^1^. (c) Platform-based multilateralism. Traditional I-U-R collaborations are predominantly bilateral and project-bound. DAST alliances require multi-party data interoperability across heterogeneous digital platforms (IoT devices, satellite systems, farm management software), necessitating novel standards and API governance mechanisms.

#### Partner selection for innovation alliance

**Fig. 1:**
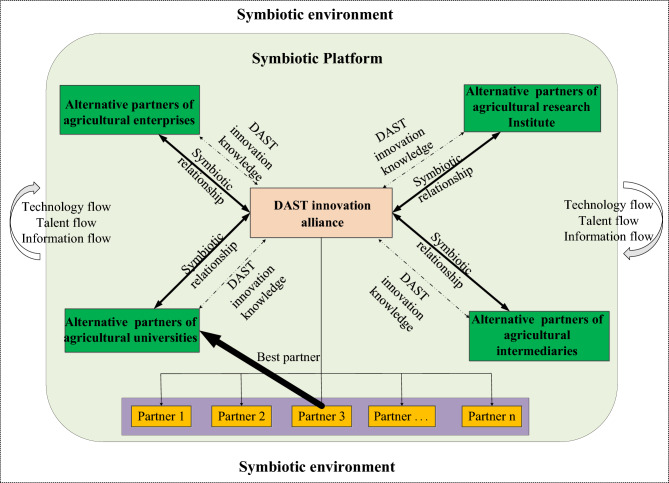
Theoretical model of partner selection for DAST innovation alliance.

Drawing upon existing research, this study posits that a DAST innovation alliance functions as a collaborative entity where risks and rewards are collectively shared. Within such alliances, digital agriculture firms, academic institutions, and research organizations collaborate on technological and product development to access mutually beneficial agricultural resources. The formation and longevity of such strategic innovation alliances fundamentally rely on enduring partnerships built on trust, with partner compatibility emerging as a critical factor in maintaining alliance stability^24,25^. Figure 1 illustrates the conceptual framework governing partner selection processes within digital agricultural science and technology innovation alliances.

Within the digital agri-tech innovation consortium, agricultural enterprises, academic institutions, research organizations, and intermediary agencies constitute the core innovation actors. Collaborative interactions involving knowledge transfer, technological exchange, and information sharing between agricultural corporations and academic institutions significantly enhance the consortium’s innovative capacity, as these partnerships facilitate the integration of diverse organizational competencies and specialized knowledge systems^26,27^. Through sustained collaboration in knowledge dissemination, talent cultivation, and technological advancement, innovation participants gradually evolve into mutually beneficial relationships that enable comprehensive sharing of complementary digital agricultural resources^28,29^. The establishment of effective symbiotic partnerships fundamentally depends on members’ capability to identify appropriate collaborators with aligned strategic objectives^30,31^. While the consortium’s operational framework necessitates selecting technical collaborators from multiple innovation entities, this partner selection process involves intricate decision-making dynamics influenced by evolving technological requirements and market conditions^32,33^.

#### MCDM problems and field theory

Field theory constitutes a fundamental concept in physics, emphasizing the integrated and continuous nature of spatial interactions that demonstrate imperceptible reciprocal relationships and dynamic influences^34,35^. This theoretical framework finds extensive applications across disciplines such as chemistry, materials science, biology, and medical research. Roe et al.^36^ systematically elaborated decision field theory while extending its implementation to diverse decision-making contexts. Song et al.^37^ innovatively integrated hesitation fuzzy sets (HFS) with decision field theory (DFT) to establish a novel group decision approach termed hesitation fuzzy decision field theory (HFDFT) . Lee and Son^38^ subsequently merged the DFT paradigm with the DeGroot model to create the advanced DFT-L decision architecture. From the perspective of procedural decision analysis, Liu et al.^39^ formulated a multi-attribute group decision methodology for handling incomplete linear ordered ranking (ILOR) information based on decision field principles . The current study’s application of field theory diverges from conventional decision field approaches, offering critical insights into understanding resource complementarity, rational allocation, and compatibility optimization during partner selection processes.

Significant progress has been achieved in scholarly investigations regarding partner selection for industrial strategic alliances. These studies have predominantly centered on two key dimensions: determinants affecting partnership choices and methodological frameworks for evaluation. Regarding influential factors, extensive analyses have examined dimensions such as trust^40^, resource complementarity^41^, and collaborative compatibility^42,43^ in shaping alliance formation decisions. Furthermore, academic explorations have integrated theoretical lenses including knowledge management paradigms, synergy principles, and game theory models to indirectly examine partner selection dynamics through collaborative innovation motivations and incentive mechanisms. Notably, certain researchers have employed resource dependence theory to investigate how alliance partner selection and knowledge acquisition strategies impact innovation outcomes^44^, while others have developed selection frameworks emphasizing project accountability and historical partnership performance^45^. In methodological advancements, researchers have developed and refined diverse analytical frameworks, such as the DEMATEL-AEW-FVIOR hybrid approach^46^, an integrated fuzzy MCDM framework combining LBWA (Level Based Weight Assessment) and Complex Proportional Assessment (COPRAS) method^47^ and a hybrid Fuzzy Analytic Hierarchy Process—Combined Compromise Solution AHP-CoCoSo) model^48^. In addition, researchers have introduced novel decision-making frameworks. For instance, Qiu et al.^49^ proposed two-stage model employs the integration of Step-Wise Weight Assessment Ratio Analysis (SWARA) and Weighted Aggregated Sum Product Assessment (WASPAS) under spherical fuzzy (SF) conditions to address the strategic sequencing of sustainable policies. Similarly, Chang et al.^50^ proposed a fuzzy delphi method integrated with cluster analysis. Golui et al.^51^ presented a novel correlation coefficient for (FFSs), which incorporates the hesitancy function into the calculation for the first time. Bera et al.^52^ presented a multi-criteria group decision-making (MCGDM) approach by combining neutrosophic information with the Technique for Order of Preference by Similarity to Ideal Solution (TOPSIS) to solve the MSME location selection issue. Current research limitations reveal that evaluations typically occur at isolated temporal points through static analytical lenses, prioritizing optimal partner identification over alliance evolution. Notably, limited attention has been given to phased evaluations across multiple timeframes or dynamic partnership adjustments involving member transitions. Moreover, existing scholarship insufficiently addresses the longitudinal nature of alliance partnerships and the cumulative effects of iterative interactions between innovation networks and their constituent members.

Therefore, this research develops a dynamic partner selection framework for DAST innovation alliances by integrating dynamic intuitionistic fuzzy decision-making principles with field theory, emphasizing the evolving nature of collaborative innovation processes. The paper’s structure is organized into the following sections: section "Empirical research" formulates an alliance partner selection mechanism through field theory applications, analyzing the logical consistency and sustainability when evaluating potential collaborators while establishing key withdrawal criteria for existing alliance members. Section "Discussion" demonstrates practical implementation through China’s 5G Agricultural Digitalization Alliance case study, verifying the proposed methodology’s operational viability and practical value. The final section (section "Conclusion") synthesizes research findings, discusses theoretical contributions and managerial insights, and outlines potential directions for future investigation.

### Research framework

This multiple attribute decision-making (MADM) problem involves two partner panels: alternative cooperation innovation partners and the partners in an established DAST innovation alliance. The set of the partners in the established DAST innovation alliance is denoted as $RT{\text{ = \{ }}RT_{1} ,RT_{2} , \cdots ,RT_{m} {\mathrm{\} }}$, and the set of the alternative cooperation innovation partners is denoted as $S=\left\{ {S_{1} ,S_{2} ,...,S_{m} } \right\}$. The selection of partners for the DAST innovation alliance is a MADM problem, and the criteria set is represented as $G = \left\{ {G_{1} ,G_{2} ,...,G_{n} } \right\}$,$T = \left\{ {t_{1} ,t_{2} ,...,t_{k} } \right\}$, which is a set of decision stages composed of $k$ decision periods. In different decision periods $t_{k}$, the attribute value of the $S_{i}$ scheme under the attribute $G_{j}$ is represented by an intuitionistic fuzzy number, denoted as $\alpha_{ij}^{k} = (\mu_{ij}^{k} ,\upsilon_{ij}^{k} )$. Hence, the intuitionistic fuzzy decision matrix of a decision period $t_{k}$ can be expressed as:

$$\begin{gathered} A(t_{k} ) = (\alpha_{ij}^{k} )_{m \times n} = (\mu_{ij}^{k} ,\upsilon_{ij}^{k} )_{m \times n} = \\ \left[ {\begin{array}{*{20}c} {(\mu_{11}^{k} ,\upsilon_{11}^{k} )} & {(\mu_{12}^{k} ,\upsilon_{12}^{k} )} & {...} & {(\mu_{1n}^{k} ,\upsilon_{1n}^{k} )} \\ {(\mu_{21}^{k} ,\upsilon_{21}^{k} )} & {(\mu_{22}^{k} ,\upsilon_{22}^{k} )} & \ldots & {(\mu_{2n}^{k} ,\upsilon_{2n}^{k} )} \\ \vdots & \vdots & \ddots & \vdots \\ {(\mu_{m1}^{k} ,\upsilon_{m1}^{k} )} & {(\mu_{m2}^{k} ,\upsilon_{m2}^{k} )} & \ldots & {(\mu_{mn}^{k} ,\upsilon_{mn}^{k} )} \\ \end{array} } \right] \\ \end{gathered}$$

The weight of attributes in different time periods is different. The weight vector of attributes in the $$k$$ time period is unknown, denoted as $$\omega^{k} = (\omega_{1}^{k} ,\omega_{2}^{k} ,...,\omega_{n}^{k} )^{T}$$, where $$\omega_{j}^{k} \in [0,1],\sum\limits_{j = 1}^{n} {\omega_{j}^{k} } = 1$$. In this paper, the grey relational degree method and intuitionistic fuzzy entropy method are combined to solve the attribute weight. The time weight vector is also unknown, denoted as $$\eta_{{t_{k} }} = (\eta (t_{1} ),\eta (t_{2} ),...,\eta (t_{p} ))^{T}$$, which reflects the importance attached to different time periods in the decision-making process, where $$\eta (t_{k} ) \in [0,1]$$$$,\sum\limits_{k = 1}^{p} {\eta (t_{k} )} = 1$$, $$\eta (t_{k} )$$ is the weight of the $$k$$th time period. In this paper, based on the principle of maximum entropy, a nonlinear programming model is established to solve the temporal weight vector. Then the orthogonal projection method is used to evaluate the alternatives comprehensively. In the specific DAST innovation, the complementary resource set between the members and alternative members of the innovation alliance is expressed as *YR* = (*YR*_1_ … *YR*_*l*_). Then, based on the complementary resource a field theory model is integrated to select the DAST partner.

**Fig. 2:**
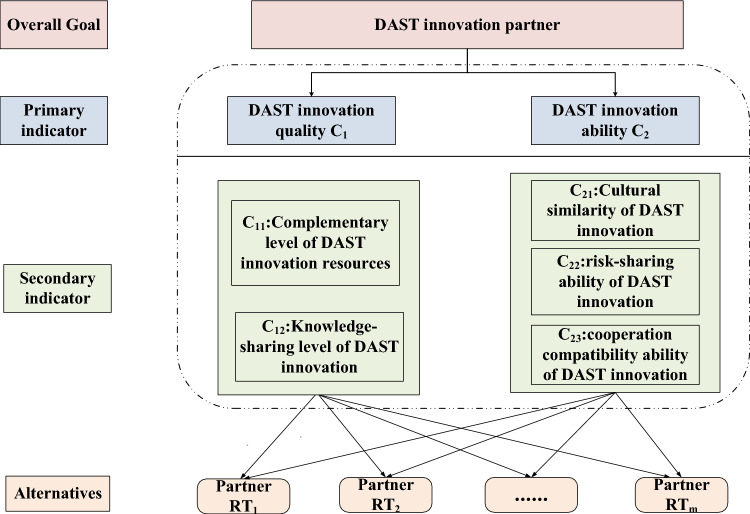
The index framework of partner selection for DAST innovation alliance based on the field theory.

To establish the evaluation framework for DAST alliance partner selection, the concepts of static attributes and dynamic competencies derived from field theory are incorporated to assess collaborative innovation potential. From the perspective of field theory, prospective alliance members exhibit dual dimensions: inherent qualities representing their stable characteristics, and developmental capabilities indicating their potential for integration into DAST innovation networks. The selection process emphasizes three critical dimensions: resource synergy, collaborative knowledge transfer, and organizational alignment^53^. Through synthesis of existing scholarship and analysis of DAST alliance characteristics, the partner evaluation system comprises five core criteria: resource complementary level of a partner^54,55^, knowledge-sharing level^53,56^, the cultural similarity of a partner^57,58^, and risk-sharing ability^59^, cooperation compatibility ability^60^. Figure 2 shows the partner selection index framework of the DAST innovation alliance based on the above theoretical framework.

### Preliminary

#### Intuitionistic fuzzy sets (IFSs)

##### Definition 1

^61^Let a set $X = \{ x_{1} ,x_{2} , \cdots x_{n} \}$ be a finite universe of discourse, then the set $A = \{ < x,u_{A} (x),v_{A} (x)|x \in X > \}$ is an intuitionistic fuzzy set, where, $u_{A} (x)$ and $v_{A} (x)$ are the membership degree and non-membership degree of the element $x$ in $X$ belonging to $A$, respectively: $\mu_{A} (x):X \to \left[ {0,1} \right]$,$\upsilon_{A} (x):X \to \left[ {0,1} \right]$, and $0 \le \mu_{A} (x) + \upsilon_{A} (x) \le 1,x \in X$.Besides, $\pi_{A} = 1 - \mu_{A} (x) - \upsilon_{A} (x)$ is the degree of indeterminacy of $x$ in $X$ belonging to $A$.

##### Definition 2

^61^Let $\alpha_{1} = (\mu_{{\alpha_{1} }} ,\nu_{{\alpha_{1} }} )$ and $\alpha_{2} = (\mu_{{\alpha_{2} }} ,\nu_{{\alpha_{2} }} )$ be two IFNs, $\lambda$ be a real number, and $\lambda > 0$, then

1. $$\alpha_{1} \oplus \alpha_{2} = (\mu_{{\alpha_{1} }} + \mu_{{\alpha_{2} }} - \mu_{{\alpha_{1} }} \mu_{{\alpha_{2} }} ,\nu_{{\alpha_{1} }} \nu_{{\alpha_{2} }} );$$
2. $$\alpha_{1} \otimes \alpha_{2} = (\mu_{{\alpha_{1} }} \mu_{{\alpha_{2} }} ,\nu_{{\alpha_{1} }} + \nu_{{\alpha_{2} }} - \nu_{{\alpha_{1} }} \nu_{{\alpha_{2} }} );$$
3. $$\lambda \alpha_{1} = (1 - (1 - \mu_{{\alpha_{1} }} )^{\lambda } ,\nu_{{\alpha_{1} }}^{\lambda } );$$
4. $$\alpha_{{1}}^{\lambda } = \left( {\mu_{{\alpha_{{1}} }}^{\lambda } ,1 - \left( {1 - \nu_{{\alpha_{{1}} }}^{{}} } \right)^{\lambda } } \right).$$

##### Definition 3

^62^Let $\alpha_{j} = (\mu_{{A_{j} }} (x),\nu_{{A_{j} }} (x)),j = 1,2,...,n,$ be an IFN, then

$$IFWA_{\omega } (\alpha_{1} ,\alpha_{2} ,...,\alpha_{n} ) = \sum\limits_{j = 1}^{n} {\omega_{j} \alpha_{j} = (1 - \prod\limits_{j = 1}^{n} {\left( {1 - \mu_{{A_{j} }} (x)} \right)^{{\omega_{j} }} } } ,\prod\limits_{j = 1}^{n} {\nu_{{A_{j} }} (x)^{{\omega_{j} }} } )$$

is an intuitionistic fuzzy weighted averaging (IFWA) operator. Where, $IFWA:Q^{n} \to Q$,$\omega { = }\left( {\omega_{1} \omega_{2} ...,\omega_{n} } \right)^{T} ,\omega_{j} \ge 0$,$j{ = }1,2,...,n$,$\sum\nolimits_{j = 1}^{n} {\omega_{j} } = 1.$

##### Definition 4

^63^Let $X = \left\{ {x_{1} ,x_{2} ,...,x_{n} } \right\}$ be a finite universe of discourse and $T = \left\{ {t_{1} ,t_{2} ,...,t_{p} } \right\}$ be a discrete time set. A dynamic intuitionistic fuzzy set (DIFS) $\tilde{A}$ in $X$ is defined as: $\tilde{A} = \left\{ {\left\langle {x,u_{{\tilde{A}}} (x,t),v_{{\tilde{A}}} (x,t)} \right\rangle \left| {x \in X,t \in T} \right.} \right\}$. Where $u_{{\tilde{A}}} (x,t):X \times T \to [0,1]$ denotes the membership function, $v_{{\tilde{A}}} (x,t):X \times T \to [0,1]$ denotes the non-membership function, satisfying the condition:$0 \le u_{{\tilde{A}}} (x,t) + v_{{\tilde{A}}} (x,t) \le 1,\forall x \in X,t \in T$, the hesitation margin at time t is defined as:$\pi_{{\tilde{A}}} (x,t) = {1 - }u_{{\tilde{A}}} (x,t){ - }v_{{\tilde{A}}} (x,t)$, representing the degree of uncertainty or indeterminacy evolving over time.

##### Definition 5

^63^Let *t* be a timing variable, then $\alpha (t) = \left( {\mu_{\alpha (t)} (x),\nu_{\alpha (t)} (x)} \right)$ is an IFN, where $\mu_{\alpha (t)} (x) \in \left[ {0,1} \right],\nu_{\alpha (t)} (x) \in \left[ {0,1} \right]$,$\mu_{\alpha (t)} (x){ + }\nu_{\alpha (t)} (x) \le 1$, if $t{ = }t_{1} t_{2} ...,t_{n}$, and $\alpha (t_{1} ),\alpha (t_{2} ),...,\alpha (t_{p} )$ is defined as p IFSs with different periods.

##### Definition 6

^63^Let $$\alpha_{{t_{k} }} = (\mu_{{t_{k} }} ,\nu_{{t_{k} }} )$$$$\left( {k{ = }1,2,...,p} \right)$$ be an IFN at period $$t_{k}$$, and $$\eta (t_{k} ) = (\eta (t_{1} )\eta (t_{2} )...,\eta (t_{p} ))^{T}$$ be the weight vector of the time period $$t_{k}$$, then

$$DIFWG_{\eta (t)} (\alpha_{{t_{1} }} ,\alpha_{{t_{2} }} ,...,\alpha_{{t_{p} }} ) = \prod\limits_{k = 1}^{p} {\alpha_{{t_{k} }}^{{\eta (t_{k} )}} } = (\prod\limits_{k = 1}^{p} {\mu_{{t_{k} }}^{{\eta (t_{k} )}} } ,1 - \prod\limits_{k = 1}^{p} {(1 - \nu_{{t_{k} }} } )^{{\eta (t_{k} )}} )$$

is a dynamic intuitionistic fuzzy weighted (DIFWG) operator, where, $$\eta (t_{k} ) \in \left[ {0,1} \right],\sum\nolimits_{k = 1}^{p} {\eta (t_{k} )} = 1$$$$\left( {k{ = }12...,p} \right).$$

##### Definition 7

^63^Let $\alpha_{1} = (\mu_{{\alpha_{1} }} ,\nu_{{\alpha_{1} }} )$ and $\alpha_{2} = (\mu_{{\alpha_{2} }} ,\nu_{{\alpha_{2} }} )$ be two IFSs, then the Euclidean distance of the two IFSs is:

$$d(\alpha_{1} ,\alpha_{2} ) = \sqrt[{}]{{\frac{1}{2}\left[ {\left( {\mu_{{\alpha_{1} }} - \mu_{{\alpha_{2} }} } \right)^{2} + \left( {\nu_{{\alpha_{1} }} - \nu_{{\alpha_{2} }} } \right)^{2} + \left( {\pi_{{\alpha_{1} }} - \pi_{{\alpha_{2} }} } \right)^{2} } \right]}}$$

where $$\pi_{{\alpha_{i} }} = 1 - \mu_{{\alpha_{i} }} - \nu_{{\alpha_{i} }} ,(i = 1,2).$$

#### Determination of attribute weights

In the fuzzy multi-attribute decision-making framework, establishing reasonable attribute weights plays a pivotal role in ensuring decision quality. This study proposes an integrated weighting technique that combines gray correlation analysis with the maximum deviation approach to determine attribute significance. The hybrid methodology simultaneously considers decision-makers’ experiential judgments and quantitative data characteristics, effectively mitigating potential biases from subjective assumptions while preventing over-reliance on information system outputs. This methodology balances decision-makers’ subjective preferences with the objectives derived from decision-related data, thereby enhancing the robustness of weight determination through complementary analytical perspectives.

Grey relational analysis addresses decision-making challenges under conditions of incomplete information and limited data points. This methodology assesses interconnections among system variables while quantifying their mutual impacts. The technique’s foundation, as outlined by Wei^64^, examines geometric similarities in data sequence curves from experimental samples to determine relational proximity.

The positive and negative ideal solutions for each attribute at time period $k$ can be expressed as $A_{j}^{ + k} = (\alpha_{1}^{ + k} ,\alpha_{2}^{ + k} ,...,\alpha_{n}^{ + k} )$ and $A_{j}^{ - k} = (\alpha_{1}^{ - k} ,\alpha_{2}^{ - k} ,...,\alpha_{n}^{ - k} )$, respectively, where

1 $$\alpha_{j}^{ + k} = (\mu_{j}^{ + k} ,\upsilon_{j}^{ + k} ) = (\mathop {max}\limits_{1 \le i \le m} \mu_{ij}^{k} ,\mathop {min}\limits_{1 \le i \le m} \upsilon_{ij}^{k} ),(j = 1,2,...,n)$$

2 $$\alpha_{j}^{ - k} = (\mu_{j}^{ - k} ,\upsilon_{j}^{ - k} ) = (\mathop {min}\limits_{1 \le i \le m} \mu_{ij}^{k} ,\mathop {max}\limits_{1 \le i \le m} \upsilon_{ij}^{k} ),(j = 1,2,...,n)$$

According to Definition 6, in time period $k$, the distance between a solution and its correlated positive ideal solution is $d_{ij}^{ + } (A_{ij}^{k} ,A_{j}^{ + k} )$, and the distance between a solution and its correlated negative ideal solution is $d_{ij}^{ - } (A_{ij}^{k} ,A_{j}^{ - k} )$. Then, the correlation coefficients of the $i$ th solution with the positive and negative ideal solutions under attribute $j$ in time period $k$ can be calculated as follows:

3 $$\varepsilon_{ij}^{ + k} = \frac{{\mathop {min}\limits_{i} \mathop {min}\limits_{j} d_{ij}^{ + } (A_{ij}^{k} ,A_{j}^{ + k} ) + \rho \mathop {max}\limits_{i} \mathop {max}\limits_{j} d_{ij}^{ + } (A_{ij}^{k} ,A_{j}^{ + k} )}}{{d_{ij}^{ + } (A_{ij}^{k} ,A_{j}^{ + k} ) + \rho \mathop {max}\limits_{i} \mathop {max}\limits_{j} d_{ij}^{ + } (A_{ij}^{k} ,A_{j}^{ + k} )}}$$

4 $$\varepsilon_{ij}^{ - k} = \frac{{\mathop {min}\limits_{i} \mathop {min}\limits_{j} d_{ij}^{ - } (A_{ij}^{k} ,A_{j}^{ - k} ) + \rho \mathop {max}\limits_{i} \mathop {max}\limits_{j} d_{ij}^{ - } (A_{ij}^{k} ,A_{j}^{ - k} )}}{{d_{ij}^{ - } (A_{ij}^{k} ,A_{j}^{ - k} ) + \rho \mathop {max}\limits_{i} \mathop {max}\limits_{j} d_{ij}^{ - } (A_{ij}^{k} ,A_{j}^{ - k} )}}$$

where $\rho$ is the distinguishing coefficient and $\rho \in [0,1]$, and generally, $\rho$ is set to 0.5.

$\chi^{k} = (\chi_{1}^{k} ,\chi_{2}^{k} ,...,\chi_{m}^{k} )$ is defined as the attribute weight vector in time period $k$, and $\chi_{j}^{k} \in [0,1],\sum\limits_{j = 1}^{m} {\chi_{j}^{k} } = 1(k = 1,2,...,p)$. As the correlation coefficient vector of a solution and its positive ideal solution at time period $k$ is $(1,1,...,1)$, the sum of composite deviations of the correlation degree between the $i$ th solution and its positive ideal solution in time period $k$ is

5 $$\gamma_{i}^{k} (\chi^{k} ) = \sum\limits_{j = 1}^{m} {[(1 - \varepsilon_{ij}^{ + k} )} \chi_{j}^{k} ]^{2}$$

Hence, a nonlinear programming model, (M–1), can be constructed to minimize the sum of composite deviations of the correlation degree between all solutions and their correlated positive ideal solutions:

$$(M - 1)\left\{ {\begin{array}{*{20}c} {min\gamma^{k} (\chi^{k} ) = \sum\limits_{i = 1}^{n} {\sum\limits_{j = 1}^{m} {[(1 - \varepsilon_{ij}^{ + k} )} \chi_{j}^{k} ]^{2} } } \\ {s.t.\sum\limits_{j = 1}^{m} {\chi_{j}^{k} } = 1(k = 1,2,...,p)} \\ \end{array} } \right.$$

The solution of (M–1) is

6 $$\left\{ {\begin{array}{*{20}l} {\begin{array}{*{20}l} {\lambda = - [\sum\limits_{j = 1}^{m} {(\sum\limits_{i = 1}^{n} {(1 - \varepsilon_{ij}^{ + k} )^{2} )^{ - 1} ]^{ - 1} } } } \hfill \\ {\chi_{j}^{k} = [\sum\limits_{j = 1}^{m} {(\sum\limits_{i = 1}^{n} {(1 - \varepsilon_{ij}^{ + k} )^{2} )^{ - 1} ]^{ - 1} } } \times [\sum\limits_{i = 1}^{n} {(1 - \varepsilon_{ij}^{ + k} )^{2} ]^{ - 1} } } \hfill \\ \end{array} } \hfill \\ \end{array} } \right.$$

On the other hand, taking the difference of decision-making information under time series characteristics and the preference of decision-makers toward each attribute in different periods into account, the intuitionistic fuzzy entropy method is used to determine the weight of each attribute under time series^63^.

In time period $t_{k}$, the intuitionistic fuzzy entropy of the attribute is

7 $$E_{j}^{k} = \frac{1}{m}\sum\limits_{i = 1}^{m} {\left\{ {1 - \sqrt {\left( {1 - \pi_{ij}^{k} } \right)^{2} - \mu_{ij}^{k} \nu_{ij}^{k} } } \right\}}$$

Set $\phi_{j}^{k}$ as the attribute weight of the time period $t_{k}$, an optimization model of attribute weight in the time period $t_{k}$, (M–2), is established as follows:

$$(M - 2)\left\{ {\begin{array}{*{20}c} {\min \sum\limits_{j = 1}^{n} {\left( {\phi_{j}^{k} } \right)^{2} } E_{j}^{k} } \\ {s.t\sum\limits_{j = 1}^{n} {\phi_{j}^{k} = 1} } \\ \end{array} } \right.$$

After solving model (M–2), the weight of the attribute can be obtained:

8 $$\phi_{j}^{k} = \frac{{\left( {E_{j}^{k} } \right)^{ - 1} }}{{\sum\limits_{j = 1}^{n} {\left( {E_{j}^{k} } \right)^{ - 1} } }}$$

Therefore, by combining the grey correlation analysis method and the intuitionistic fuzzy entropy method, the weight vector of comprehensive attributes can be expressed as Eq. (9).

9 $$\omega_{j} (t_{k} ) = \eta \chi_{j}^{k} { + }(1 - \eta )\phi_{j}^{k} (k = 1,2,...,p)$$

where $\omega_{j} (t_{k} )$ is the weight vector of comprehensive attributes in the time period of $k$, and $\omega_{j} (t_{k} )$ captures the competitive priority of evaluation dimension j in the alliance’s current lifecycle stage. Usually, weight distribution patterns diagnose alliance strategic orientation and decision-makers identify resource allocation misalignment.$\eta (0 \le \eta \le 1)$ is the experience factor. Generally, $\eta { = }0.5$.

#### Determination of time weights

##### Definition 8

^65^Let $\theta = \sum\limits_{k = 1}^{p} {\frac{p - k}{{p - 1}}\eta (t_{k} )}$, and $\theta \in [0,1]$, then $\theta$ is the time degree of time sequence weight vector: $\eta_{{t_{k} }} = (\eta (t_{1} ),\eta (t_{2} ),...,\eta (t_{p} ))^{T}$. $\eta (t_{k} )$ represents the strategic relevance intensity of historical information for current decision-making. A higher $\eta (t_{k} )$ indicates that period t’s alliance performance carries greater learning value for predicting future trajectories.

Time degree reflects a decision-maker’s preference degree towards time series, and the decision-makers generally give $\theta$ value based on experience and preference. When $\theta$ trends to 0, it indicates that the decision-maker prefers recent information; when θ tends to 1, it means that the decision-maker prefers past information.

Information entropy quantifies the temporal weight vector’s capacity to assimilate information volume. Higher entropy values correspond to diminished information content, with this relationship being mathematically represented through Eq. (10).

10 $$f(\eta (t_{k} )) = - \sum\limits_{k = 1}^{p} {\eta (t_{k} )\ln \eta (t_{k} ),k = 1} ,2,...,p$$

According to Definition 8, $\eta_{{t_{k} }} = (0,0,...,1)^{T}$, $\theta = 0$ indicates a decision-maker completely prefers recent information, and the positive ideal time weight vector is denoted as $\eta_{{t_{k} }}^{ + } = (0,0,...,1)^{T}$;$\eta_{{t_{k} }} = (1,0,...,0)^{T}$,$\theta = 1$ indicates a decision-maker completely prefers past information, and the negative ideal time weight vector is denoted as $\eta_{{t_{k} }}^{ - } = (1,0,...,0)^{T}$.

The distance between the two time weight vectors, $\underline{\eta }_{{t_{k} }} = (\underline{\eta } (t_{1} ),\underline{\eta } (t_{2} ),...,\underline{\eta } (t_{p} ))^{T}$ and $\overline{\eta }_{{t_{k} }} = (\overline{\eta }(t_{1} ),\overline{\eta }(t_{2} ),...,\overline{\eta }(t_{p} ))^{T}$, is set to be

11 $$d(\underline{\eta }_{{t_{k} }} ,\overline{\eta }_{{t_{k} }} ) = \sqrt {\sum\limits_{k = 1}^{p} {(\underline{\eta } (t_{k} ) - \overline{\eta }(t_{k} ))^{2} } }$$

The separation between a temporal weight vector $\eta_{{t_{k} }} = (\eta (t_{1} ),\eta (t_{2} ),...,\eta (t_{p} ))^{T}$ and its associated positive ideal temporal weight vectors, along with the interval between the weight vector and the related negative ideal temporal weight vectors, can be determined as follows:

12 $$d(\eta_{{t_{k} }} ,\eta_{{t_{k} }}^{ + } ) = \sqrt {\sum\limits_{k = 1}^{p - 1} {\eta (t_{k} )^{2} + (1 - \eta (t_{p} ))^{2} } }$$

13 $$d(\eta_{{t_{k} }} ,\eta_{{t_{k} }}^{ - } ) = \sqrt {(1 - \eta (t_{1} ))^{2} + \sum\limits_{k = 2}^{p} {\eta (t_{k} )^{2} } }$$

Hence, a nonlinear multi-objective programming model, (M-3), is constructed by maximizing the ability of information uptake, maximizing the closeness degree to positive ideal time weight vector, and minimizing the closeness degree to the negative ideal time weight vector:

$$(M - 3)\left\{ {\begin{array}{*{20}l} {max\begin{array}{*{20}c} {} \\ \end{array} f(\eta (t_{k} )) = - \sum\limits_{k = 1}^{p} {\eta (t_{k} )\ln \eta (t_{k} )} } \hfill \\ \begin{gathered} max\begin{array}{*{20}c} {} \\ \end{array} d(\eta_{{t_{k} }} ,\eta_{{t_{k} }}^{ - } ) = \sqrt {(1 - \eta (t_{1} ))^{2} + \sum\limits_{k = 2}^{p} {\eta (t_{k} )^{2} } } \hfill \\ min\begin{array}{*{20}c} {} \\ \end{array} d(\eta_{{t_{k} }} ,\eta_{{t_{k} }}^{ + } ) = \sqrt {\sum\limits_{k = 1}^{p - 1} {\eta (t_{k} )^{2} + (1 - \eta (t_{p} ))^{2} } } \hfill \\ \end{gathered} \hfill \\ {s.t.{\kern 1pt} {\kern 1pt} {\kern 1pt} {\kern 1pt} \theta = \sum\limits_{k = 1}^{p} {\frac{p - k}{{p - 1}}\eta (t_{k} ),{\kern 1pt} {\kern 1pt} \sum\limits_{k = 1}^{p} {\eta (t_{k} ) = 1,\eta (t_{k} ) \in \left[ {0,1} \right],k = 1,2,..,p} } } \hfill \\ \end{array} } \right.$$

To simplify the calculation, this multi-objective optimization model is transformed into a single-objective optimization model, (M-4):

$$(M - 4)\left\{ {\begin{array}{*{20}l} {min\begin{array}{*{20}c} {} \\ \end{array} g = c\sqrt {\sum\limits_{k = 1}^{p - 1} {\eta (t_{k} )^{2} + (1 - \eta (t_{p} ))^{2} } } - c\sqrt {(1 - \eta (t_{1} ))^{2} + \sum\limits_{k = 2}^{p} {\eta (t_{k} )^{2} } } + (1 - 2c)(\sum\limits_{k = 1}^{p} {\eta (t_{k} )\ln \eta (t_{k} )} )} \hfill \\ {s.t.{\kern 1pt} {\kern 1pt} {\kern 1pt} {\kern 1pt} \theta = \sum\limits_{k = 1}^{p} {\frac{p - k}{{p - 1}}\eta (t_{k} ),{\kern 1pt} {\kern 1pt} \sum\limits_{k = 1}^{p} {\eta (t_{k} ) = 1,\eta (t_{k} ) \in \left[ {0,1} \right],k = 1,2,..,p} } } \hfill \\ \end{array} } \right.$$

where $c$ is the equilibrium coefficient, $c \in [0,0.5]$. Model (M-4) is solved using Lingo 11.0 software, and a time series weight vector, $\eta_{{t_{k} }} = (\eta (t_{1} ),\eta (t_{2} ),...,\eta (t_{p} ))^{T}$, is obtained.

#### Orthogonal projection method

The orthogonal projection method was proposed by Hua et al.^66^ in 2004. In this method, a vertical distance is defined as the distance between planes whose normal vectors are the lines connecting the positive and negative ideal solutions, respectively, between the positive and negative ideal solutions^66^. This main content is to overcome the limitations of the Euclidean distance in the TOPSIS method, serving as a replacement for the Euclidean distance to determine the closeness of alternatives.

**Fig. 3:**
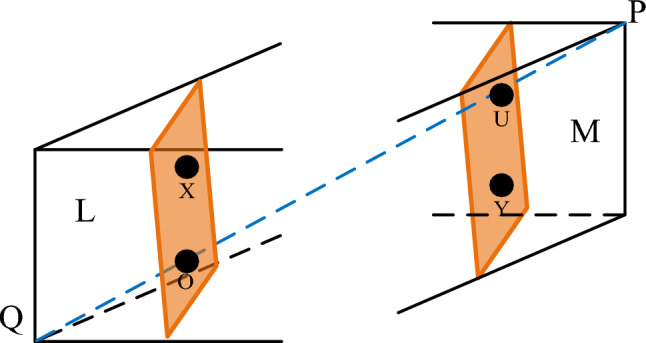
Vertical distance in three-dimensional space.

To establish a vivid description of the vertical distance, the three-dimensional space is taken as an example (see Fig. 3). P and $\mathrm{Q}$ are the relatively positive and negative ideal schemes among all alternatives. The distance between the plane passes through the $\mathrm{X}$ point with the straight line $\mathrm{PQ}$ as the normal vector and the plane passes the $\mathrm{Y}$ point with the straight line $\mathrm{PQ}$ as the normal vector, that is, the distance between the planes $\mathrm{L}$ and $\mathrm{M}$. This distance is the Euclidean distance between the orthogonal projection points $\mathrm{O}$ and $\mathrm{U}$ on the line $\mathrm{PQ}$ of points $\mathrm{X}$ and $\mathrm{Y}$. Suppose the vectors corresponding to points $\mathrm{P}$, $\mathrm{Q}$, $\mathrm{X}$, $\mathrm{Y}$ are $\mathrm{p}$, $\mathrm{q}$, $\mathrm{x}$, $\mathrm{y}$; then, the vertical distance between points $\mathrm{X}$ and $\mathrm{Y}$ is

14 $$V = \frac{{\left| {(p - q) \cdot (x - y)} \right|}}{{\left\| {p - q} \right\|}}$$

In the formula, $\cdot$ is the point multiplication of the vector; $| |$ is the absolute value; $\parallel \parallel$ is the norm.

**Fig. 4:**
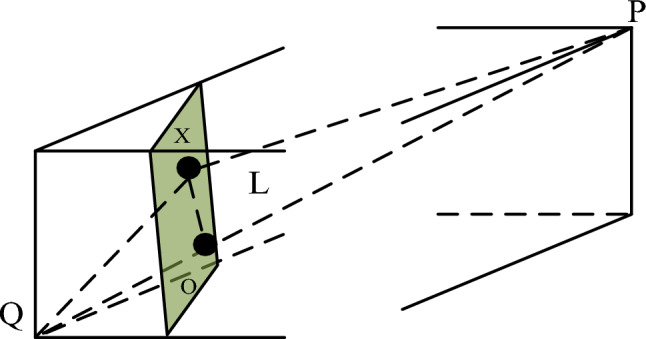
Vertical distance from any point X to the ideal solution point.

When both points X and Y are non-ideal solutions, the vertical distance between them can be calculated using the vector translation method in formula (14). If either point is an ideal solution, the vertical distance from any point X to the ideal solution point H or K can be determined. As shown in Fig. 4, in addition to the vector translation method, we propose a more straightforward algorithm.

As illustrated in Fig. 4, the concept of vertical distance refers to the straight-line distance from any point X to the ideal solution point P or Q. Taking the vertical distance from point X to the ideal solution point Q as an example, we derive the formula. Since the line XO is perpendicular to the line PQ, we obtain Eq. (15):

15 $$\left\{ {\begin{array}{*{20}c} {\left( {QX} \right)^{2} - \left( {QO} \right)^{2} = \left( {XP} \right)^{2} - \left( {OP} \right)^{2} } \\ {QO + OP = PQ} \\ \end{array} } \right.$$

The distance between point O and point Q is the vertical distance from X to the ideal solution point Q, expressed as:

16 $$QO = \frac{{\left( {QX} \right)^{2} + \left( {PQ} \right)^{2} - \left( {XP} \right)^{2} }}{2PQ}$$

Similarly, the distance from point O to point P is the vertical distance from X to the ideal solution point P, expressed as:

17 $$OP = \frac{{\left( {XP} \right)^{2} + \left( {PQ} \right)^{2} - \left( {XQ} \right)^{2} }}{2PQ}$$

In this paper, the orthogonal projection method is used to rank the schemes, and the basic steps are as follows.

**Step 1:** The normalized matrix of the benefit attributes and the normalized matrix of the cost attributes are constructed as follows:

18 $$\overline{u}_{ij}^{k} = \frac{{\mu_{ij}^{k} }}{{\sum\limits_{{\text{i = 1}}}^{m} {\upsilon_{ij}^{k} } }},\overline{\upsilon }^{k}_{ij} = \frac{{\upsilon_{ij}^{k} }}{{\sum\limits_{{\text{i = 1}}}^{m} {\mu_{ij}^{k} } }},i \in 1,2,...,m;j \in I_{1} ,{\text{benefit attribute}}$$

19 $$\overline{u}_{ij}^{k} = \frac{{{1 \mathord{\left/ {\vphantom {1 {\upsilon_{ij}^{k} }}} \right. \kern-0pt} {\upsilon_{ij}^{k} }}}}{{{1 \mathord{\left/ {\vphantom {1 {\sum\limits_{{\text{i = 1}}}^{m} {\mu_{ij}^{k} } }}} \right. \kern-0pt} {\sum\limits_{{\text{i = 1}}}^{m} {\mu_{ij}^{k} } }}}},\overline{\upsilon }_{ij}^{k} = \frac{{{1 \mathord{\left/ {\vphantom {1 {\mu_{ij}^{k} }}} \right. \kern-0pt} {\mu_{ij}^{k} }}}}{{{1 \mathord{\left/ {\vphantom {1 {\sum\limits_{{\text{i = 1}}}^{m} {\upsilon_{ij}^{k} } }}} \right. \kern-0pt} {\sum\limits_{{\text{i = 1}}}^{m} {\upsilon_{ij}^{k} } }}}},i \in 1,2,...,m;j \in I_{2} ,{\text{cost attribute}}$$

**Step 2:** Determine the attribute weight $\omega_{j} (t_{k} )$ of different time periods using Model (M-1), Model (M-2), and Eq. (9). Then, the weighted intuitionistic fuzzy matrix, $\hat{A}(t_{k} ) = (\hat{A}_{ij}^{k} )_{m \times n} = (\omega_{j}^{k} \hat{A}_{ij}^{k} )_{m \times n}$, is obtained. Model (M-4) is used to solve the time series weight vector, $\eta_{{t_{k} }} = (\eta (t_{1} ),\eta (t_{2} ),...,\eta (t_{p} ))^{T}$, and the dynamic intuitionistic fuzzy weighted geometry (DIFWG) operator in Definition 6 is used to aggregate the weighted intuitionistic fuzzy decision matrix of each time period, and the dynamic intuitionistic fuzzy comprehensive decision matrix $\hat{A}(t_{k} ) = (\hat{A}_{ij}^{k} )_{m \times n}$ is obtained, where,

$$\begin{gathered} R = (r_{ij}^{{}} )_{m \times n} = (\sigma_{ij}^{{}} ,\tau_{ij}^{{}} )_{m \times n} = \\ \left[ {\begin{array}{*{20}c} {(\sigma_{11}^{{}} ,\tau_{11}^{{}} )} & {(\sigma_{12}^{{}} ,\tau_{12}^{{}} )} & {...} & {(\sigma_{1n}^{{}} ,\tau_{1n}^{{}} )} \\ {(\sigma_{21}^{{}} ,\tau_{21}^{{}} )} & {(\sigma_{22}^{{}} ,\tau_{22}^{{}} )} & \ldots & {(\sigma_{2n}^{{}} ,\tau_{2n}^{{}} )} \\ \vdots & \vdots & \ddots & \vdots \\ {(\sigma_{m1}^{{}} ,\tau_{m1}^{{}} )} & {(\sigma_{m2}^{{}} ,\tau_{m2}^{{}} )} & \ldots & {(\sigma_{mn}^{{}} ,\tau_{mn}^{{}} )} \\ \end{array} } \right] \\ \end{gathered}$$

**Step 3:** The positive and negative ideal solutions of the dynamic intuitionistic fuzzy synthetic decision matrix $R = (r_{ij} )_{m \times n}$ is determined as follows:

20 $$r_{j}^{ + } = (\sigma_{j}^{ + } ,\tau_{j}^{ + } ){ = }(\mathop {max}\limits_{1 \le i \le m} \sigma_{ij} ,\mathop {min}\limits_{1 \le i \le m} \tau_{ij} ),(j = 1,2,...,n)$$

21 $$r_{j}^{ - } = (\sigma_{j}^{ - } ,\tau_{j}^{ - } ){ = }(\mathop {min}\limits_{1 \le i \le m} \sigma_{ij} ,\mathop {max}\limits_{1 \le i \le m} \tau_{ij} ),(j = 1,2,...,n)$$

**Step 4:** Definition 7 is utilized to calculate the distance between every intuitionistic fuzzy number $r_{ij}$ and its corresponding positive and negative ideal solutions (Eq. 22 and Eq. 23, respectively) and the distance between the positive ideal solution and the negative ideal solution (Eq. 24):

22 $$d(r_{{^{i} }} ,r^{ + } ) = \sqrt {\frac{1}{2}\sum\limits_{j = 1}^{n} {\left[ {(\sigma_{{_{j} }}^{ + } - \sigma_{ij} )^{2} + (\tau_{{_{j} }}^{ + } - \tau_{ij} )^{2} + (\pi_{{_{j} }}^{ + } - \pi_{ij} )^{2} } \right]} }$$

23 $$d(r_{{^{i} }} ,r^{ - } ) = \sqrt {\frac{1}{2}\sum\limits_{j = 1}^{n} {\left[ {(\sigma_{{_{j} }}^{ - } - \sigma_{ij} )^{2} + (\tau_{{_{j} }}^{ - } - \tau_{ij} )^{2} + (\pi_{{_{j} }}^{ - } - \pi_{ij} )^{2} } \right]} }$$

24 $$d(r^{ + } ,r^{ - } ) = \sqrt {\frac{1}{2}\sum\limits_{j = 1}^{n} {\left[ {(\sigma_{{_{j} }}^{ - } - \sigma_{{_{j} }}^{ + } )^{2} + (\tau_{{_{j} }}^{ - } - \tau_{{_{j} }}^{ + } )^{2} + (\pi_{{_{j} }}^{ - } - \pi_{{_{j} }}^{ + } )^{2} } \right]} }$$

**Step 5:** The vertical distance between each scheme and the negative ideal solution is calculated by using Eq. (25).

25 $$V_{i}^{ - } = \frac{{d^{2} (r^{ + } ,r^{ - } ) + d^{2} (r_{i} ,r^{ - } ) - d^{2} (r_{i} ,r^{ + } )}}{{2d(r^{ + } ,r^{ - } )}}$$

The schemes can be ranked according to the vertical distance. The larger the vertical distance $V_{i}^{ - }$, the farther the scheme $S_{i}$ is from the negative ideal solution and the better the scheme is $S_{i}$.

### Proposed approach based on field theory

#### Concept and definition of field model

In physics, the field theory reflects an invisible interaction and influence effect in a holistic and continuous manner^67^. In the innovation progress of an DAST innovation alliance, the relationships between the alliance and its partners also have a certain holistic characteristic, and the relationship between the two parties has a continuity characteristic. Therefore, this paper adopts the field theory to investigate the selection strategy of cooperative innovation partners for an DAST innovation alliance.

The field source (denoted as $O$) constitutes a DAST innovation alliance, characterized by its capacity to leverage innovative assets throughout the collaborative development process. As established by^67^, the alliance’s collaborative innovation performance directly correlates with its innovative asset base, quantified through two key metrics: resource deployment efficiency and reserve quantities. The inventory of innovation resources is defined as $M{ = }(m_{1} {,}m_{2} {,} \ldots {,}m_{n} )$, where $n$ represents the spatial dimension of innovation resources. Any innovation resource should satisfy the condition: $0 \le m_{i} \le 1$. When $m_{i} { = }1$, it indicates that this innovation resource meets the need of cooperative innovation, and when $m_{i} { = 0}$, it indicates that this innovation resource does not meet the needs of cooperative innovation. The utilization rate of innovation resources is defined as $P{ = }(p_{1} {,}p_{2} {,} \ldots {,}p_{n} )$, and $0 \le p_{i} \le 1$, where $p_{i}$ represents the utilization of the resource stock $m_{i}$. The supply of innovation resources will change dynamically in different time periods due to the internal factors of DAST innovation and the external environment.

Therefore, when incorporating temporal considerations into the analysis framework (with the study period defined as $T{ = }(t_{1} {,}t_{2} {,} \ldots {,}t_{n} )$), the collaborative innovation capability level of the DAST innovation alliance, denoted as $Q_{T}$, can be mathematically represented through the following formulation:

26 $$Q_{T} = M \times P_{T} = \sum\limits_{i = 1}^{n} {m_{i} p_{i} }$$

During the cooperative innovation process of an DAST innovation alliance, there is resource complementarity between the candidate partners and the innovation alliance. Setting the resource inventory of the candidate partner as $H{ = }(h_{1} {,}h_{2} {,} \ldots {,}h_{n} )$, the demand for the resources as $\overline{M}$, and the space saturation degree as $\tilde{M}{ = (}\overbrace {{{1,1,}...{,1}}}^{n})$, the cooperative innovation capability quality of the candidate partner, $q_{T}$, can be expressed as:

27 $$q_{T} { = (}(\tilde{M} \oplus \overline{M}) \cap H) \times P_{T} = \sum\limits_{i = 1}^{n} {[(1 \oplus m_{i} )} \wedge h_{i} ]p_{i}$$

The intensity of the collaborative innovation capability field describes the magnitude of influence exerted by field sources on the DAST innovation alliance within specific regions of this field. Field strength exhibits an inverse relationship with distance from the source, where greater proximity corresponds to stronger field effects. The directional vector of this collaborative innovation capability field, denoted as $E_{T}$, originates at potential partners and terminates at the field source. $E_{T}$ is determined through the formula provided below:

28 $$E_{T} { = }\frac{{K_{T} Q_{T} }}{{R_{T}^{2} }}$$

where $K_{T}$ is the cooperative innovation capability effect produced by the DAST innovation alliance and an alternative partner at different time points, and $R_{T}$ is the radius of the cooperative innovation capability at different time points. In the current collaborative innovation environment, with the recruiting of a candidate partner, the increase of the alliance’s innovation quality brought by an innovation resource, can be expressed as $I_{T}$, and $K_{T}$ can be calculated using the following equation:

29 $$K_{T} { = }\frac{{I_{T} }}{{(Q_{T} + q_{T} )}}$$

The gravitational force associated with collaborative innovation capacity signifies the level of appeal or acknowledgment that a domain source holds toward the innovative capabilities of prospective partners. This gravitational effect, denoted by $F_{T}$, is mathematically represented as:

30 $$F_{T} { = }E_{T} \times q_{T} = \frac{{I_{T} Q_{T} q_{T} }}{{(Q_{T} + q_{T} )R_{T}^{2} }}$$

The collaborative innovation capacity radius ($R$) serves as a dynamic variable that holistically evaluates potential partners’ qualifications and competencies^67^. This radius parameter is derived from assessing the candidate’s innovation potential through a dynamic intuitionistic fuzzy decision-making framework incorporating temporal dimensions and orthogonal projection analysis. The computational model integrates multidimensional evaluation criteria that quantify both static attributes and evolving capabilities within collaborative innovation systems.

Assuming the competence and capacity of the primary source is $C_{f}$, while the substitute collaborator’s proficiency and potential are $C$, the collaborative innovation radius can be mathematically expressed as follows:

31 $$R_{T} { = 1 + }C_{f} - C,C \in [0,1]$$

**Fig. 5:**
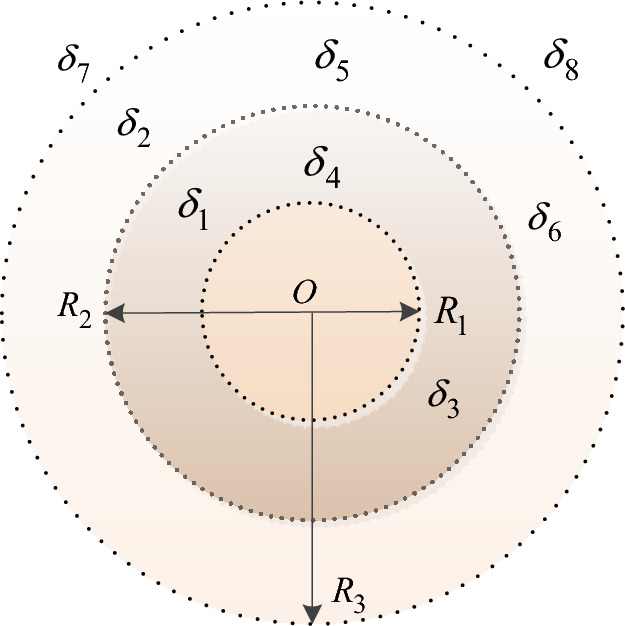
Field model of the cooperative innovation capability.

The quality and ability of $R_{1}$ location in the field is defined as $C_{1}$, $C_{f} \in [C_{1} ,1]$, and the quality and ability of an DAST innovation alliance is 1 ($C_{f} { = }1$). Then, the radius of the cooperative innovation ability is $R_{T} { = 2} - C$. To facilitate the following discussion and analysis, the field of cooperative innovation capability in this study is divided into four different sections shown in Fig. 5: $(0,R_{1} ]$ is the strong cooperative innovation capability zone, $(R_{1} ,R_{2} ]$ is the medium cooperative innovation capability zone, $(R_{2} ,R_{3} ]$ is the weak cooperative innovation capability zone, and $(R_{3} ,\infty ]$ is the non-cooperative innovation capability zone. From this Fig. 5, virtual coil represents the radiation range of field strength with different cooperative innovation ability, and $\delta_{1} ,\delta_{2} ,\delta_{3} , \cdots ,\delta_{8}$ represent the alternative partners in different circles.

The willingness resistance is the opportunity and risk cost of a candidate partner to join an DAST innovation alliance. Setting $Z_{1}$ as the opportunity cost and $Z_{2}$ as the risk cost, the willingness resistance of a candidate partner can be expressed as:

32 $$F_{TW} { = }Z_{1} + Z_{2}$$

The critical condition for a candidate partner to enter or exit the innovation alliance is as follows: the radius of cooperative innovation ability, $R_{T}$, is equal or smaller than the radius threshold $\varphi_{T}$, ($R_{T} \le \varphi_{T}$) and gravity of the cooperative innovation ability, $F_{T}$ is equal or greater than the gravity threshold $\xi_{T}$, and equal or greater than the resistance $F_{TW}$, ($F_{T} \ge \xi_{T}$ and $F_{T} \ge F_{TW}$).

#### Dynamic characteristic analysis for the partner selection of DAST innovation alliances

During the formation and evolution of DAST innovation alliances, enhancing collaborative innovation efficacy necessitates periodic partner renewal and structural adaptation. To optimize alliance performance, member composition requires dynamic refinement by phasing out underperforming collaborators while actively recruiting qualified entities that align with alliance standards. The partner selection mechanism within DAST alliances demonstrates evolving parameters, where innovation capacity metrics and domain expertise benchmarks undergo continuous recalibration based on shifting operational requirements. This necessitates establishing flexible evaluation frameworks that account for technological advancements and market fluctuations, ensuring sustained alignment between partner capabilities and alliance objectives.

Defining $N_{t}$ as the partner who withdraws from an DAST innovation alliance at the $t$th time period, the quality of the cooperative innovation capability can be expressed as:

33 $$Q_{t + 1} { = }Q_{t} + q_{t} - N_{t}$$

The dynamic change in the field strength of the innovation capability of the DAST innovation alliance in the $t{ + }1$th time period can be defined as:

34 $$E_{t + 1} = \frac{{[K_{t + 1} {(}Q_{t} + q_{t} - N_{t} )]}}{{R_{t + 1}^{2} }}$$

**Fig. 6:**
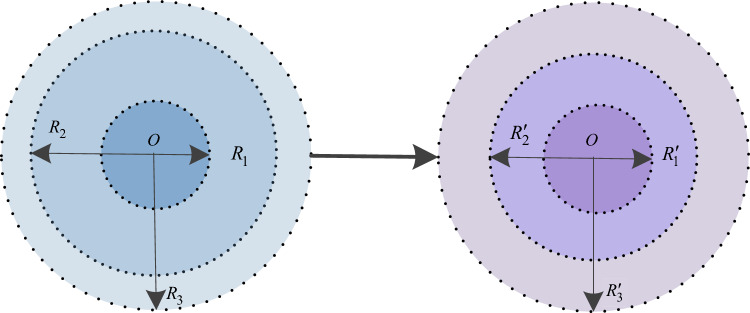
Dynamic changes of the sections of the cooperative innovation capability.

Hence, the sections of the cooperative innovation capability field can also be adjusted accordingly, as shown in Fig. 6.

**Fig. 7:**
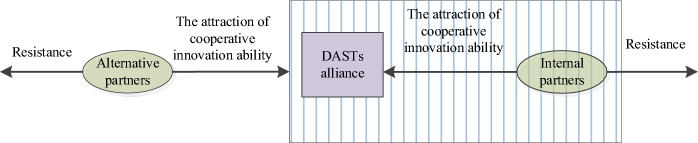
Internal and external forces of an DAST innovation alliance based on the cooperative innovation field.

The partners located in the inner sections of the cooperative innovation capability field have strong cooperative innovation capability fields and can fully integrate and use the resources of the DAST innovation alliance to promote further improvement of their cooperative innovation capabilities. With significant improvement in the level of partners’ innovation performances, their willingness resistance may change in multiple folds. The willingness resistance can be expressed as $F_{(t + 1)W} { = }\chi M_{t + 1}$^68^. A candidate partner located close to the outer section of the cooperative innovation capability field is influenced by both the cooperative innovation capability gravity, $F_{T}$, and the willingness resistance, $F_{TW}$, as shown in Fig. 7.

#### Dynamic selection procedure of DAST innovation alliances

The cooperative innovation field model is carried with the following steps:

**Step 1:** The cooperative innovation ability of each partner is obtained by the integrated fuzzy orthogonal projection approach method based on decision rules.

**Step 2:** Based on the evaluation of the quality and capabilities of the DAST innovation alliance and its candidate partners, the field strength, attraction, radius, and resistance based on innovation ability are calculated by Eqs. (28–34).

**Step 3:** The radius threshold value and attraction threshold value are calculated based on expert consultation.

**Step 4:** Based on the trigger point, one or more potential partners are eliminated. The remaining candidates are subsequently ranked according to their resultant force values, from which one or more partners are selected to join the DAST innovation alliance.

**Fig. 8:**
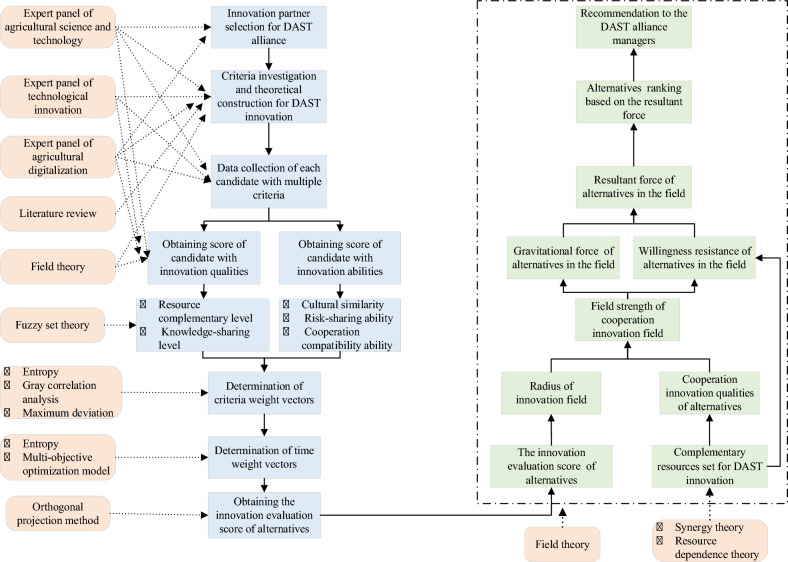
Dynamic selection procedure of DAST innovation alliance.

Figure 8 presents processes used for DAST innovation alliance partner selection. In the integrated time-weighted fuzzy orthogonal projection method, major steps include: (a) the partner selection index framework of the DAST innovation alliance is structured based on field theory; (b) cooperation aspiration of alternative partners is obtained using fuzzy set theory with innovation qualities (resource complementary level and knowledge-sharing level) and innovation abilities(cultural similarity, risk-sharing ability and cooperation compatibility ability); (c) criteria weight vectors are determined by combining gray correlation analysis with the maximum deviation approach and entropy measure method; (d) time weight vectors are also calculated by entropy measure method with a multi-objective optimization model; (e) innovation evaluation scores of alternatives are obtained using orthogonal projection method. In the cooperation innovation field model for partner selection based on field theory, major steps include: (a) complementary resources set for DAST innovation are structured by experts method; (b) the cooperation innovation ability of alternatives and complementary resources set is transformed into the radius of innovation field and the cooperation innovation qualities of alternatives, respectively; (c) field strength of the cooperation innovation field is calculated by using proposed field method; (d) gravitational force and willingness resistance of alternatives in cooperation innovation field, respectively; (e) resultant force of alternatives and alternatives ranking is obtained, and (f) one or more innovation partners are recommended to the DAST alliance managers.

## Empirical research

### Empirical background

In 2019, China Mobile Communications Corporation held the 5G + Agricultural Digitalization Conference and established the 5G Agricultural Digitalization Alliance. The initial 10 members of the alliance ($RT_{1} ,RT_{2} ,RT_{3} ,RT_{4} ,RT_{5} ,RT_{6} ,RT_{7} ,RT_{8} ,RT_{9} ,RT_{10}$) includes research institutes, universities, leading enterprises, and agent firms specialized in agricultural information services. The goal of this alliance is to promote the transformation and upgrading of traditional agriculture, to build an industry cooperation platform that integrates production, teaching, and research, and to build an ecological and intelligent agriculture infrastructure. This research takes China’s 5G Agricultural Digitalization Alliance as a case study. In order to meet the needs of the digital agricultural market and to build a long-term and stable cooperative relationship, a new agricultural digital alliance needs to be established based on the current one, including finding two highly matched partners for in-depth cooperation and eliminating partners that are not in line with digital transformation.

### Data and scenarios

In the study, the data are empirical that collected from surveys, based on the preliminary qualitative and quantitative index screening work and market research, five candidate partners were identified, denoted as $S = \{ S_{1} ,S_{2} ,S_{3} ,S_{4} ,S_{5} \}$. Then, 15 experts were randomly invited from the three related fields of technological innovation, agricultural science and technology innovation, and agricultural digitalization, the expert information is as shown in Table 1.

**Table 1:** The expert information.

No	Category	Position	Expertise
1	University	Professor	Agricultural science and technology innovation
2	University	Professor	Agricultural science and technology innovation
3	University	Professor	Agricultural science and technology innovation
4	University	Professor	Agricultural digitalization
5	University	Professor	Agricultural digitalization
6	Research Institute	Senior professional	Agricultural digitalization
7	Research Institute	Senior professional	Agricultural digitalization
8	Research Institute	Senior professional	Agricultural science and technology innovation
9	Research Institute	Senior professional	Agricultural science and technology innovation
10	Research Institute	Senior professional	technological innovation
11	Enterprise	Senior management position	Agricultural digitalization
12	Enterprise	Senior management position	Agricultural digitalization
13	Enterprise	Senior management position	Agricultural science and technology innovation
14	Enterprise	Senior management position	Technological innovation
15	Enterprise	Senior management position	Technological innovation

This study employed an expert panel consultation method to elicit professional judgments on technological innovation, agricultural science and technology innovation, and agricultural digitalization. The present study involved expert elicitation of professional judgments only, with no collection of personal health information, biometric data, or sensitive personal data. We think such minimal-risk research is exempt from ethical review.All 15 experts were recruited based on their published expertise in technological innovation, agricultural science and technology innovation, or agricultural digitalization and voluntarily participated in the study. Prior to data collection, each expert received a detailed information sheet explaining: (i) the study purpose and procedures, (ii) the voluntary nature of participation, (iii) the right to withdraw at any time, and (iv) data anonymization procedures. Verbal informed consent was obtained, as the study involved no identifiable personal data. In addition, no compensation was provided to participants. As the study involved anonymous aggregation of professional opinions with no individual attribution; written consent was impractical due to geographic dispersion and time constraints, as approved by the Ethics Committee of Northeast Forestry University.

To protect expert privacy, identifiable information (names and specific institutions) has been anonymized. Experts were selected based on: (i) minimum 10 years of experience in the three related fields; (ii) active engagement in relevant research or policy-making; (iii) representation from diverse regions and sectors (universities, research institute, and enterprise). The present study involved expert elicitation of professional judgments only, with no collection of personal health information, biometric data, or sensitive personal data. Verbal informed consent was obtained, as the study involved no identifiable personal data. In addition, no compensation was provided to participants.

These experts evaluate the following two principles: first, based on the evaluation indicators (G_1_–G_5_) for selecting DAST innovation alliance partners proposed in section “Research framework”, which takes account of the dynamic impact of time factors on the evaluation process. Each expert carried out anonymous evaluation on the 10 members of alliance and 5 alternative partners in 3 different time periods, and filled in the evaluation sheet with language information set. And the evaluation results are independent. Secondly, after the evaluation, the range of each indicator was narrowed according to the range values of each indicator in the first circle evaluation form. Then, a new evaluation sheet was returned to each expert for a second evaluation. Feedback and evaluation went through multiple cycles until the 15 experts’ evaluation results converged, and the convergence results are as showed in Appendix. During the process, we used the Kendall’s W and intraclass correlation (ICC) analysis to determine the experts’ evaluation results consistent. At the time period of T1, Kendall’s W and ICC analyses demonstrated moderate consistency in expert ratings (mean W = 0.692) and moderate reliability (mean ICC = 0.698). The convergence results were highly consistent with the expert mean, validating the effectiveness of the convergence process. Similarly, at the time periods of T2 and T3, Kendall’s W and ICC analyses demonstrated moderate to high consistency. Then, we converted the linguistic set information into the form of intuitionistic fuzzy numbers (see Table 2). Next, according to the requirements of the agricultural digital alliance for scientific and technological innovation resources, experts evaluated the 7 types of innovation resources ($YR_{1} \sim YR_{7}$) required for the scientific and technological innovation of the alliance. The evaluation results are shown in Table 3. Where "1" indicates that the innovation body has the DAST innovation resources, and "0" indicates that innovation body is lack of the DAST innovation resources. The utilization rate of DAST innovation resources is represented in "()". S_1_-S_5_ represent the five alternative partners and YR1-YR7 represents complementary resources.

**Table 2:** Intuitionistic fuzzy decision matrix of each time period.

$t_{{1}}$	$G_{1}$	$G_{2}$	$G_{3}$	$G_{4}$	$G_{5}$
$RT_{1}$	(0.5,0.3)	(0.4,0.3)	(0.5,0.2)	(0.5,0.3)	(0.5,0.4)
$RT_{2}$	(0.6,0.2)	(0.5,0.3)	(0.8,0.1)	(0.5,0.4)	(0.7,0.2)
$RT_{3}$	(0.4,0.5)	(0.6,0.3)	(0.7,0.1)	(0.4,0.3)	(0.6,0.1)
$RT_{4}$	(0.6,0.3)	(0.3,0.5)	(0.4,0.5)	(0.3,0.6)	(0.5,0.3)
$RT_{5}$	(0.2,0.7)	(0.4,0.5)	(0.5,0.3)	(0.6,0.3)	(0.4,0.5)
$RT_{6}$	(0.4,0.5)	(0.4,0.2)	(0.6,0.2)	(0.3,0.4)	(0.4,0.5)
$RT_{7}$	(0.4,0.5)	(0.5,0.4)	(0.7,0.2)	(0.4,0.5)	(0.6,0.3)
$RT_{8}$	(0.3,0.5)	(0.6,0.3)	(0.5,0.4)	(0.4,0.5)	(0.4,0.5)
$RT_{9}$	(0.5,0.3)	(0.4,0.5)	(0.4,0.3)	(0.6,0.2)	(0.3,0.6)
$RT_{10}$	(0.4,0.5)	(0.7,0.1)	(0.3,0.6)	(0.5,0.3)	(0.4,0.3)
$S_{1}$	(0.6,0.1)	(0.5,0.3)	(0.5,0.3)	(0.4,0.5)	(0.6,0.2)
$S_{2}$	(0.4,0.5)	(0.3,0.6)	(0.7,0.2)	(0.3,0.5)	(0.5,0.3)
$S_{3}$	(0.6,0.1)	(0.3,0.5)	(0.4,0.5)	(0.6,0.3)	(0.5,0.4)
$S_{4}$	(0.5,0.4)	(0.5,0.3)	(0.4,0.3)	(0.6,0.1)	(0.4,0.2)
$S_{5}$	(0.4,0.5)	(0.7,0.2)	(0.5,0.4)	(0.3,0.6)	(0.3,0.5)

$t_{2}$	$G_{1}$	$G_{2}$	$G_{3}$	$G_{4}$	$G_{5}$
$RT_{1}$	(0.6,0.1)	(0.5,0.3)	(0.3,0.5)	(0.4,0.3)	(0.5,0.4)
$RT_{2}$	(0.4,0.5)	(0.7,0.2)	(0.5,0.4)	(0.4,0.5)	(0.5,0.2)
$RT_{3}$	(0.3,0.6)	(0.5,0.3)	(0.4,0.5)	(0.6,0.3)	(0.5,0.3)
$RT_{4}$	(0.4,0.3)	(0.5,0.4)	(0.6,0.2)	(0.5,0.3)	(0.2,0.6)
$RT_{5}$	(0.4,0.5)	(0.4,0.3)	(0.3,0.6)	(0.4,0.5)	(0.6,0.1)
$RT_{6}$	(0.5,0.3)	(0.4,0.5)	(0.7,0.2)	(0.4,0.4)	(0.5,0.2)
$RT_{7}$	(0.3,0.6)	(0.6,0.1)	(0.5,0.3)	(0.5,0.4)	(0.3,0.5)
$RT_{8}$	(0.7,0.1)	(0.3,0.5)	(0.2,0.5)	(0.5,0.3)	(0.5,0.4)
$RT_{9}$	(0.3,0.5)	(0.3,0.6)	(0.6,0.3)	(0.4,0.2)	(0.4,0.5)
$RT_{10}$	(0.5,0.4)	(0.5,0.3)	(0.4,0.5)	(0.6,0.2)	(0.5,0.3)
$S_{1}$	(0.5,0.4)	(0.4,0.5)	(0.6,0.3)	(0.4,0.3)	(0.5,0.3)
$S_{2}$	(0.3,0.5)	(0.5,0.2)	(0.4,0.5)	(0.6,0.2)	(0.3,0.5)
$S_{3}$	(0.4,0.5)	(0.6,0.2)	(0.5,0.3)	(0.4,0.5)	(0.7,0.1)
$S_{4}$	(0.5,0.3)	(0.4,0.5)	(0.6,0.2)	(0.5,0.4)	(0.4,0.4)
$S_{5}$	(0.5,0.2)	(0.3,0.5)	(0.7,0.1)	(0.5,0.2)	(0.6,0.2)

$t_{3}$	$G_{1}$	$G_{2}$	$G_{3}$	$G_{4}$	$G_{5}$
$RT_{1}$	(0.3,0.5)	(0.6,0.2)	(0.4,0.5)	(0.5,0.3)	(0.4,0.3)
$RT_{2}$	(0.2,0.5)	(0.8,0.1)	(0.4,0.5)	(0.4,0.3)	(0.5,0.4)
$RT_{3}$	(0.4,0.2)	(0.4,0.5)	(0.3,0.5)	(0.5,0.3)	(0.6,0.2)
$RT_{4}$	(0.4,0.5)	(0.3,0.5)	(0.2,0.6)	(0.6,0.3)	(0.4,0.4)
$RT_{5}$	(0.5,0.4)	(0.6,0.3)	(0.4,0.3)	(0.2,0.5)	(0.3,0.5)
$RT_{6}$	(0.6,0.2)	(0.3,0.6)	(0.4,0.5)	(0.6,0.1)	(0.3,0.6)
$RT_{7}$	(0.5,0.3)	(0.6,0.1)	(0.4,0.4)	(0.6,0.3)	(0.5,0.2)
$RT_{8}$	(0.5,0.2)	(0.4,0.5)	(0.6,0.3)	(0.5,0.2)	(0.5,0.4)
$RT_{9}$	(0.6,0.3)	(0.5,0.3)	(0.5,0.4)	(0.4,0.4)	(0.7,0.1)
$RT_{10}$	(0.4,0.5)	(0.6,0.3)	(0.3,0.5)	(0.5,0.4)	(0.6,0.2)
$S_{1}$	(0.5,0.2)	(0.4,0.3)	(0.5,0.3)	(0.4,0.5)	(0.3,0.5)
$S_{2}$	(0.4,0.3)	(0.4,0.5)	(0.5,0.2)	(0.6,0.3)	(0.4,0.4)
$S_{3}$	(0.5,0.4)	(0.6,0.2)	(0.4,0.3)	(0.3,0.5)	(0.5,0.2)
$S_{4}$	(0.4,0.5)	(0.5,0.3)	(0.6,0.1)	(0.5,0.2)	(0.5,0.3)
$S_{5}$	(0.6,0.2)	(0.4,0.5)	(0.4,0.3)	(0.5,0.3)	(0.8,0.1)

**Table 3:** Agricultural technology digital innovation resources of the current partners and the alternative partners.

	$YR_{1}$	$YR_{2}$	$YR_{3}$	$YR_{4}$	$YR_{5}$	$YR_{6}$	$YR_{7}$
$RT_{1}$	1(0.65)	0(0.35)	1(0.85)	1(0.65)	0(0.15)	1(0.75)	0(0.35)
$RT_{2}$	1(0.85)	1(0.75)	0(0.15)	0(0.25)	1(0.65)	0(0.15)	1(0.75)
$RT_{3}$	1(0.85)	0(0.15)	1(0.65)	1(0.85)	0(0.25)	1(0.65)	0(0.25)
$RT_{4}$	1(0.75)	1(0.85)	0(0.35)	0(0.25)	0(0.15)	0(0.25)	0(0.15)
$RT_{5}$	1(0.65)	0(0.15)	1(0.75)	1(0.85)	0(0.35)	0(0.25)	0(0.25)
$RT_{6}$	0(0.25)	1(0.85)	0(0.15)	0(0.25)	1(0.75)	0(0.25)	1(0.85)
$RT_{7}$	1(0.75)	0(0.15)	1(0.75)	1(0.85)	0(0.35)	1(0.65)	1(0.75)
$RT_{8}$	0(0.25)	0(0.15)	0(0.35)	0(0.25)	1(0.75)	0(0.25)	1(0.65)
$RT_{9}$	1(0.75)	0(0.15)	0(0.25)	0(0.25)	0(0.35)	1(0.85)	0(0.35)
$RT_{10}$	0(0.25)	1(0.85)	0(0.35)	0(0.25)	0(0.25)	0(0.25)	1(0.65)
$S_{1}$	1(0.75)	0(0.15)	0(0.25)	1(0.85)	1(0.65)	0(0.25)	1(0.65)
$S_{2}$	0(0.25)	1(0.85)	0(0.15)	1(0.85)	1(0.75)	0(0.25)	0(0.15)
$S_{3}$	1(0.65)	0(0.25)	0(0.25)	0(0.35)	1(0.85)	0(0.15)	1(0.65)
$S_{4}$	0(0.15)	0(0.25)	1(0.65)	0(0.25)	1(0.75)	1(0.75)	1(0.85)
$S_{5}$	1(0.65)	1(0.75)	0(0.35)	1(0.85)	0(0.25)	1(0.65)	1(0.75)

### The evaluation of members and candidate partners of the alliance

Based on Model (M-1) and Model (M-2), the attribute weights, $\omega_{j} (t_{k} )$, in different time periods were obtained. According to Model (M-4), the decision maker provides the time degree $\theta { = }0.3$, on the basis of experience and preference. Lingo 11.0 software is used to solve the time sequence weights $\eta (t_{k} )$ of different indicators in different time periods with $c = 1/3$, results are as shown in Table 4.

**Table 4:** Attribute weight and time weight in each time period.

$t_{i}$	$\eta (t_{k} )$	$RT_{i}$					$S_{i}$				
		$\omega_{1} (t_{k} )$	$\omega_{2} (t_{k} )$	$\omega_{3} (t_{k} )$	$\omega_{4} (t_{k} )$	$\omega_{5} (t_{k} )$	$\omega_{1} (t_{k} )$	$\omega_{2} (t_{k} )$	$\omega_{3} (t_{k} )$	$\omega_{4} (t_{k} )$	$\omega_{5} (t_{k} )$
$t_{1}$	0.184	0.213	0.183	0.194	0.220	0.189	0.229	0.165	0.228	0.178	0.200
$t_{2}$	0.232	0.182	0.187	0.204	0.233	0.193	0.400	0.155	0.153	0.166	0.125
$t_{3}$	0.584	0.208	0.192	0.211	0.199	0.191	0.146	0.155	0.423	0.152	0.123

**Table 5:** Dynamic intuitionistic fuzzy synthetic decision matrix.

	$G_{1}$	$G_{2}$	$G_{3}$	$G_{4}$	$G_{5}$
$RT_{1}$	(0.096, 0.816)	(0.135, 0.764)	(0.097, 0.847)	(0.127, 0.777)	(0.105, 0.815)
$RT_{2}$	(0.068, 0.849)	(0.215, 0.703)	(0.130, 0.828)	(0.107, 0.810)	(0.136, 0.802)
$RT_{3}$	(0.091, 0.810)	(0.109, 0.848)	(0.095, 0.838)	(0.136, 0.777)	(0.151, 0.737)
$RT_{4}$	(0.109, 0.838)	(0.075, 0.870)	(0.071, 0.866)	(0.140, 0.807)	(0.081, 0.852)
$RT_{5}$	(0.100, 0.864)	(0.127, 0.815)	(0.097, 0.817)	(0.070, 0.852)	(0.086, 0.842)
$RT_{6}$	(0.145, 0.771)	(0.075, 0.881)	(0.133, 0.818)	(0.131, 0.722)	(0.081, 0.875)
$RT_{7}$	(0.107, 0.836)	(0.152, 0.696)	(0.124, 0.800)	(0.149, 0.807)	(0.112, 0.788)
$RT_{8}$	(0.131, 0.740)	(0.094, 0.865)	(0.120, 0.813)	(0.129, 0.764)	(0.118, 0.847)
$RT_{9}$	(0.131, 0.807)	(0.100, 0.846)	(0.134, 0.809)	(0.112, 0.786)	(0.139, 0.782)
$RT_{10}$	(0.105, 0.861)	(0.157, 0.775)	(0.077, 0.874)	(0.144, 0.795)	(0.137, 0.762)
$S_{1}$	(0.135, 0.741)	(0.081, 0.847)	(0.197, 0.703)	(0.079, 0.882)	(0.064, 0.884)
$S_{2}$	(0.090, 0.826)	(0.077, 0.883)	(0.190, 0.687)	(0.116, 0.831)	(0.065, 0.886)
$S_{3}$	(0.127, 0.818)	(0.114, 0.806)	(0.150, 0.729)	(0.071, 0.885)	(0.101, 0.808)
$S_{4}$	(0.109, 0.850)	(0.096, 0.847)	(0.214, 0.590)	(0.110, 0.787)	(0.079, 0.852)
$S_{5}$	(0.143, 0.763)	(0.082, 0.881)	(0.178, 0.675)	(0.093, 0.839)	(0.134, 0.796)

Then, based on attribute weights and intuitionistic fuzzy weighted decision matrices in different time periods, the dynamic intuitionistic fuzzy weighted geometry (DIFWG) operator is used to aggregate intuitionistic fuzzy decision matrices of each time period to obtain the dynamic intuitionistic fuzzy synthetic decision matrix, as shown in Table 5.

Next, we use Eqs. (18)–(25) to determine the vertical distance between each scheme and the corresponding negative ideal solution, which reflects the innovation ability of each partner and each candidate partner of the alliance.

$$\begin{aligned} V_{{S_{i} }} & = (V_{{S_{1} }} ,V_{{S_{2} }} ,V_{{S_{3} }} ,V_{{S_{4} }} ,V_{{S_{5} }} ) \\ & = (0.{\text{0763, 0}}.0694,0.{\text{0768, 0}}.0811,0.{\mathrm{0796}}), \\ \end{aligned}$$

$$\begin{aligned} V_{{RT_{i} }} & = (V_{{RT_{1} }} ,V_{{RT_{2} }} ,V_{{RT_{3} }} ,V_{{RT_{4} }} ,V_{{RT_{5} }} ,V_{{RT_{6} }} ,V_{{RT_{7} }} ,V_{{RT_{8} }} ,V_{{RT_{9} }} ,V_{{RT_{{10}} }} ) \\ & = (0.{\text{1489, 0}}.1689,0.{\text{1368, 0}}.0533,0.{\text{0735, 0}}.1087,0.{\text{1835, 0}}.1235,0.{\text{1252, 0}}.1325). \\ \end{aligned}.$$

### The dynamic partner selection based on field model

**Step1**: Assess the comprehensive innovation capabilities of the current members and candidate partners of the alliance.

**Step2**: Use Eqs. (26) and (27) to solve the quality of the cooperative innovation capability of the alliance. The calculation results are shown in Table 6.

**Table 6:** Strength $E_{T}$, radius $R_{T}$, gravity $F_{T}$ and resistance $F_{TW}$ of the cooperative innovation ability of the current partners and the candidate partners of the alliance.

	$RT_{1}$	$RT_{2}$	$RT_{3}$	$RT_{4}$	$RT_{5}$	$RT_{6}$	$RT_{7}$	$RT_{8}$	$RT_{9}$	$RT_{10}$
$Q_{T}$	0.7733	0.8451	0.8219	0.5818	0.6923	0.7313	0.8824	0.5283	0.5424	0.5263
$E_{T}$	0.2031	0.2268	0.2131	0.1382	0.1679	0.1840	0.2407	0.1350	0.1389	0.1358
$R_{T}$	1.8511	1.8311	1.8632	1.9467	1.9265	1.8913	1.8165	1.8765	1.8748	1.8675
$F_{T}$	0.2031	0.2268	0.2131	0.1382	0.1679	0.1840	0.2407	0.1350	0.1389	0.1358
$F_{TW}$	0.0773	0.0845	0.0822	0.0582	0.0692	0.0731	0.0882	0.0528	0.0542	0.0526
$F$	0.1258	0.1423	0.1309	0.0800	0.0987	0.1109	0.1524	0.0822	0.0846	0.0832

	$S_{1}$	$S_{2}$	$S_{3}$	$S_{4}$	$S_{5}$
$q_{T}$	0.8169	0.7538	0.6825	0.8219	0.8588
$E_{T}$	0.1987	0.1820	0.1661	0.2009	0.2096
$R_{T}$	1.9237	1.9306	1.9232	1.9189	1.9204
$F_{T}$	0.1376	0.1261	0.1150	0.1391	0.1451
$F_{TW}$	0.0817	0.0754	0.0683	0.0822	0.0859
$F$	0.0559	0.0507	0.0468	0.0569	0.0593

**Step3:** Rank the current partners and the candidate partners in the alliance:

First, set the radius threshold ($\varphi_{T}$) and solve the gravitational threshold ($\xi_{T}$). Based on the previous studies^68^ and the suggestions from the expert panel, the radius threshold is set to $\varphi_{T} { = }1.94$, and the gravitational threshold of the collaborative innovation field of the candidate partners is $\xi_{T} = 0.1208$. Based on Eqs. (28)–(32), the field strength ($E_{T}$), the radius size ($R_{T}$), the gravity ($F_{T}$) and the resistance ($F_{TW}$) of the cooperation innovation ability of the current partners and the candidate partners are calculated and listed in Table 6.

**Fig. 9:**
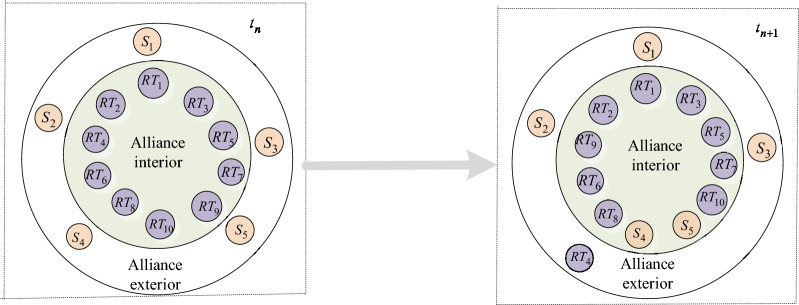
Changes in the status of the 5G agricultural digitalization alliance.

Next, several candidate partners are eliminated based on the thresholds, and new partners are selected to join the alliance according to their cooperative innovation occasion force $F$. Specific steps are as follows:

I. Screening based on the radius of the cooperative innovation capability field of the alliance $R_{T}.$ As shown in Table 6, the cooperative innovation capability field radiuses of the current partner $RT_{4}$, $R_{T} RT_{4} = 1.9467 > \phi_{T} = 1.94$, hence, $RT_{4}$ is eliminated; For the candidate partners are all less than the threshold value $\varphi_{T} { = }1.94$ and all lie in the cooperative innovation capability circle $(1,1.94].$
II. Screening based on the cooperative innovation gravity of the alliance $F_{T}$. The cooperative innovation gravity of each alternative partner $F_{T}$ is obtained. As shown in Table 6, $F_{T} (S_{3} ) = 0.1150 < \xi_{T} = 0.1208$. Hence, $S_{3}$ is eliminated; the cooperative innovation capacities of the current partners are all greater than the threshold $\xi_{T} = 0.1208$. Hence, they all enter the next screening step.
III. Final selection based on the resultant force $F$ of the cooperative innovation gravity $F_{T}$ and the cooperative innovation willingness resistance $F_{TW}$. This study fully considers the resistance $F_{TW}$ of the current partners and the candidate partner of the alliance to ensure the quality of the new partners. As the resistance of a partner is positively correlated with its own resource^68^, To ensure the quality of the partners joining the alliance, the willing resistance of each partner is set to be proportional to the amount of their own resources (set to account for 10% of the complementary resources of collaborative innovation). That is, $F_{TW} { = }\chi M_{T},$ $\chi = {0}{\mathrm{.1}},$ the resistance and its resultant force are obtained and shown in Table 6. The results show that the cooperative innovation gravities of the alliance’s current partners and candidate partners are all greater than the resistances, indicating the current partners and the candidate partners are all qualified as the partners of the alliance. The ranking of the cooperative innovation capabilities of the alternative partners is $S_{5} > S_{4} > S_{1} > S_{2}$. Therefore, the alliance has undergone a dynamic partner selection process, and members within the alliance have been adjusted: the previous partner $RT_{4}$ has been eliminated and the new partners, $S_{4}$ and $S_{{5}}$, have joined. This dynamic process is shown in Fig. 9.

### Comparative analysis

**Fig. 10:**
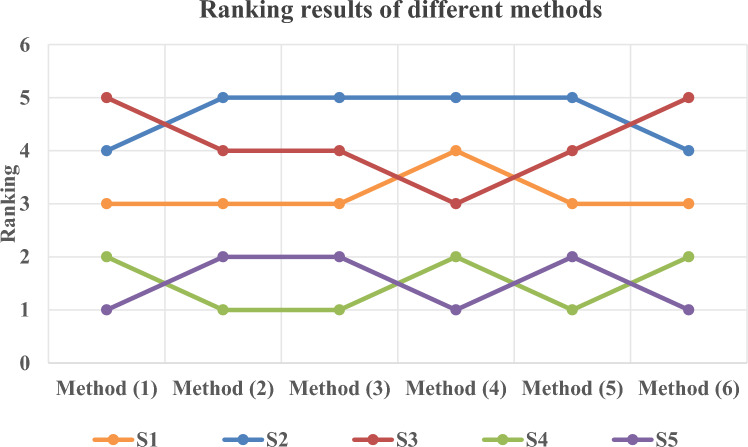
Ranking results of alternative partners of the 5G agricultural digitalization alliance.

This paper selects the scoring function, the intuitionistic fuzzy TOPSIS method and orthogonal projection method to make static selection decisions^69,70^. Additionally, two new dynamic methods are constructed by integrating the scoring function and the field theory and by integrating the TOPSIS method and the field theory, and the results of the two new dynamic methods are compared with the dynamic method proposed in this study to assess their results. The results are shown in Table 7 and Fig. 10.

**Table 7:** Results of different evaluation methods.

Static selection methods	Method (1): Scoring function		Method (2): TOPSIS		Method (3): Orthogonal projection	
	Result	Rank	Result	Rank	Result	Rank
$S_{1}$	– 0.6541	3(N)	0.404	3(N)	0.0763	3(N)
$S_{2}$	– 0.6722	4(N)	0.298	5(N)	0.0694	5(N)
$S_{3}$	– 0.6745	5(N)	0.378	4(N)	0.0768	4(N)
$S_{4}$	– 0.6068	2(Y)	0.569	1(Y)	0.0811	1(Y)
$S_{5}$	– 0.5536	1(Y)	0.534	2(Y)	0.0796	2(Y)

Dynamic selection method	Method (4): Scoring function and field theory		Method (5): TOPSIS and field theory		Method (6): The proposed method	
	Result	Rank	Result	Rank	Result	Rank
$RT_{1}$	0.0188	4(Y)	0.2355	4(Y)	0.1258	4(Y)
$RT_{2}$	0.0224	2(Y)	0.2892	2(Y)	0.1423	2(Y)
$RT_{3}$	0.0205	3(Y)	0.2409	3(Y)	0.1309	3(Y)
$RT_{4}$	0.0111	10(N)	0.1121	10(N)	0.0800	10(N)
$RT_{5}$	0.0136	6(Y)	0.1453	8(Y)	0.0987	6(Y)
$RT_{6}$	0.0176	5(Y)	0.1912	5(Y)	0.1109	5(Y)
$RT_{7}$	0.0243	1(Y)	0.3104	1(Y)	0.1524	1(Y)
$RT_{8}$	0.0132	8(Y)	0.1440	9(N)	0.0822	9(N)
$RT_{9}$	0.0136	7(Y)	0.1497	7(Y)	0.0846	7(Y)
$RT_{10}$	0.0129	9(N)	0.1555	6(Y)	0.0832	8(Y)
$S_{1}$	– 0.0094	4(N)	0.1182	3(N)	0.0559	3(N)
$S_{2}$	– 0.0096	5(N)	0.0869	5(N)	0.0507	4(N)
$S_{3}$	– 0.0088	3(N)	0.0934	4(N)	0.0468	5(N)
$S_{4}$	– 0.0068	2(Y)	0.1678	1(Y)	0.0569	2(Y)
$S_{5}$	– 0.0038	1(Y)	0.1632	2(Y)	0.0593	1(Y)

The three conventional static methods employed in this section (the scoring function, TOPSIS method, and the orthogonal projection method) do not eliminate the existing partners of the alliance, and they all select the best candidate partner to join the alliance. As shown in Table 6 and Fig. 10, the three methods show a similar ranking results, which suggests that the evaluation results of the three methods are consistent, and the selected partners are $S_{4}$ and $S_{{5}}$. The ranking result obtained with the dynamic selection proposed in this study is $S_{5} > S_{4} > S_{1} > S_{3} > S_{2}$ for the candidate partners and $RT_{7} > RT_{2} > RT_{3} > RT_{1} > RT_{6} > RT_{5} > RT_{9} > RT_{10} > RT_{8} > RT_{4}$ for the current partners, which suggest selecting $S_{4}$ and $S_{{5}}$ to join the alliance and eliminating two current partners, $RT_{4}$ and $RT_{8}$. To evaluate the proposed method, another comparative analysis is conducted, two new dynamic methods are constructed by integrating the scoring function and the field theory and by integrating the TOPSIS method and the field theory, respectively. Both methods output the similar ranks for the candidate patterns, respectively, $S_{5} > S_{4} > S_{3} > S_{1} > S_{2}$ and $S_{{4}} > S_{{5}} > S_{{1}} > S_{{3}} > S_{2}$. For the ranking of the current partners of the alliance, the results obtained with the integration of the scoring function and the field theory is $RT_{7} > RT_{2} > RT_{3} > RT_{1} > RT_{6} > RT_{5} > RT_{9} > RT_{10} > RT_{8} > RT_{4}$, and the results obtained with the integration of the TOPSIS method and the field theory is $RT_{7} > RT_{2} > RT_{3}$ $> RT_{1} > RT_{6}$ $> RT_{{{10}}} > RT_{{9}} >$ $RT_{{5}} > RT_{{8}} > RT_{4}$. Although the evaluation rankings obtained with the three different dynamic selection methods have slightly different, the final evaluation results are the same: adding $S_{4}$ and $S_{{5}}$ as new partners and removing $RT_{4}$ and $RT_{8}$. The consistency of the results is attributed to the comprehensive consideration of the quality and capabilities of the existing partners in the alliance and the resource complementarity among the partners of the alliance. Specifically, although the quality, the capabilities, and the quality of the innovative capabilities of the candidate partner $S_{5}$ are low, the complementary of the cooperative innovation resources between $S_{5}$ and the alliance and the quality of innovation capacity of $S_{5}$ is higher than that of the candidate partner $S_{4}$. Hence, the ranking result is S_5_> S_4_. These results show that the proposed method can fully take the internal innovation resources of DAST innovation alliances and the complementarity resources of the candidate partners into account to dynamically select the innovation partners of the alliance, realizing the "survival of the fittest" of the partners of the alliance. On the other hand, this study uses “vertical distance” instead of the Euclidean distance to rank the candidate partners^71,72^, which can effectively overcome the shortcomings in the TOPSIS method^73,74^, the optimal scheme which is closest to the positive ideal solution and farthest from the negative ideal solution can be obtained, which can more comprehensively reflect the closeness between the decision scheme and the ideal solution, and make the ranking result more scientific and reasonable.

### Sensitivity analysis

Additionally, different parameter values will also affect the optimal ranking results. Therefore, in the numerical example of this paper, we conduct sensitivity analysis on the ranking of optimal solutions for various parameter values.

**Fig. 11:**
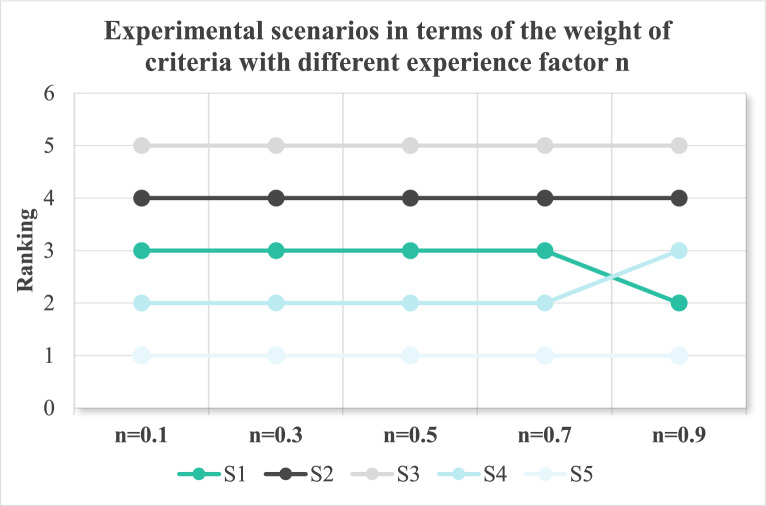
Ranking of innovation partners when n varies from 0.1 to 0.9.

By adjusting the experience factor n, the determined weights of grey relational analysis and intuitionistic fuzzy entropy show some differences; however, the overall ranking order of alternatives remains relatively consistent, and the ranking of innovation partners when n varies from 0.1 to 0.9 is as showed in Fig. 11. When n = 0.1, 0.3, 0.5, and 0.7, the ranking order of alternatives is $S_{5} > S_{4} > S_{{1}} > S_{{2}} > S_{{3}}$, while when n = 0.9, the ranking order of alternatives is $S_{5} > S_{{1}} > S_{{4}} > S_{{2}} > S_{{3}}$. The ranking results of alternatives under different n values are generally consistent with good stability, demonstrating the rationality and effectiveness of the proposed method.

**Fig. 12:**
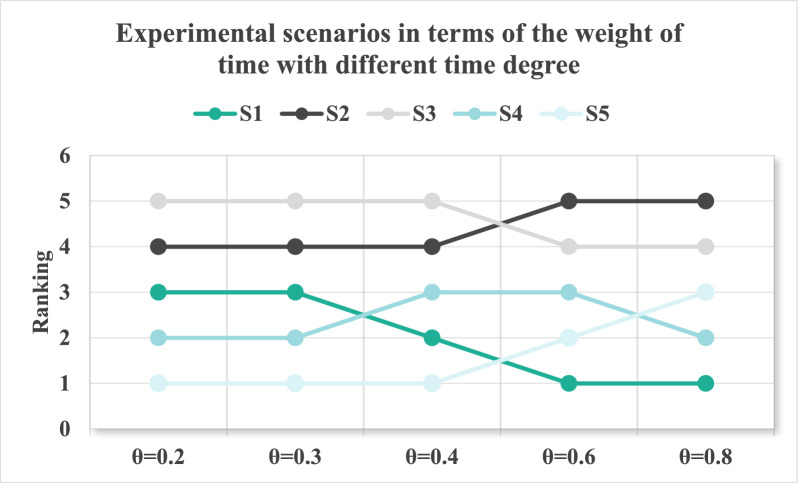
Ranking of innovation partners when θ varies from 0.2 to 0.8.

By adjusting the time degree θ, the determined weights of time show some differences; however, the overall ranking order of alternatives remains relatively consistent, and the ranking of innovation partners when n varies from 0.2 to 0.8 is as showed in Fig. 12. When θ = 0.2, 0.3, the ranking order of alternatives is $S_{5} > S_{4} > S_{{1}} > S_{{2}} > S_{{3}}$, while when θ = 0.4, the ranking order of alternatives is $S_{5} > S_{{1}} > S_{{4}} > S_{{2}} > S_{{3}}$. when θ = 0.6, the ranking order of alternatives is $S_{{1}} > S_{{5}} > S_{{4}} > S_{{3}} > S_{{2}}$. when θ = 0.8, the ranking order of alternatives is $S_{{1}} > S_{{4}} > S_{{5}} > S_{{3}} > S_{{2}}$. The sensitivity analysis reveals that the proposed model exhibits acceptable robustness across varying balance coefficients. While the time weights differ with θ, the ranking of innovation partners remains stable within low (θ ≤ 0.3) and high (θ ≥ 0.6) preference regions. A critical transition occurs at θ = 0.4, indicating that decision-makers with moderate time preferences should carefully calibrate this parameter. The consistency of rankings at extreme values (θ = 0.2 vs. θ = 0.8) suggests that strategic partner selection is more sensitive to the relative weighting of time periods than to absolute temporal focus.

**Fig. 13:**
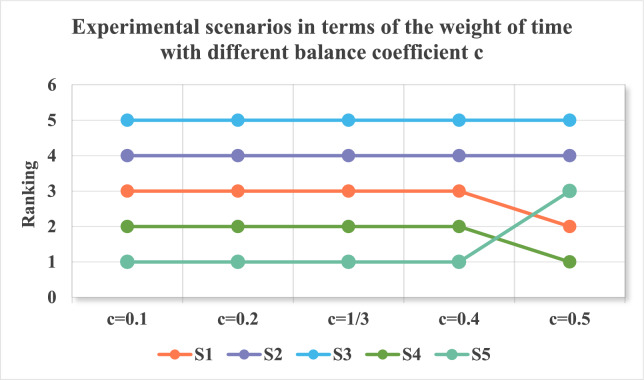
Ranking of innovation partners when c varies from 0.1 to 0.5.

By adjusting the balance coefficient c, the determined weights of time show some differences; however, the overall ranking order of alternatives remains relatively consistent, and the ranking of innovation partners when c varies from 0.1 to 0.5 is as showed in Fig. 13. When c = 0.1, 0.2, 1/3, and 0.4, the ranking order of alternatives is $S_{5} > S_{4} > S_{{1}} > S_{{2}} > S_{{3}}$, while when c = 0.5, the ranking order of alternatives is $S_{{4}} > S_{{1}} > S_{{5}} > S_{{2}} > S_{{3}}$. The ranking results of alternatives under different c values are generally consistent with good stability, demonstrating the rationality and effectiveness of the proposed method.

## Discussion

### Theoretical contributions

This research developed an integrated assessment framework for evaluating members within digital agricultural technology innovation alliances, systematically incorporating critical elements including resource synergy, collaborative knowledge exchange, cultural alignment, joint risk management, and cooperative adaptability. Furthermore, this study introduces a novel co-evolution framework that explicitly models the bidirectional relationship between alliance evaluation and alliance evolution. Unlike existing studies that treat temporal dynamics as exogenous inputs, we conceptualize alliance evolution as an endogenous process driven by evaluation-feedback loops. The study theoretically enhances collaborative innovation models and partner selection mechanisms by advancing the field theory framework, incorporating resource complementarity considerations and fuzzy set information processing within digital agricultural alliance contexts.

By applying field theory, this investigation analyzes the resource synergy, logical coherence, and compatibility dynamics within digital agricultural innovation partner selection processes, proposing an innovative analytical framework to optimize alliance member selection. By combining fuzzy set theory, grey correlation analysis method, entropy weight method and other evaluation methods, and combining the idea of combinational weighting and multi-objective optimization method, the weight and time weight of multiple attributes are calculated by double combinational weighting, and the cooperative innovation ability of multiple alternative alliance members is comprehensively evaluated by vertical projection,, addressing the limitation that traditional dynamic fuzzy TOPSIS may produce counterintuitive results when alliance performance fluctuates asymmetrically around ideal solutions. On this basis, a decision field model considering the complementarity of resources is established to select the best alliance members. Finally, the case analysis results show that the new partner selection model constructed can be applied to the partner selection management of digital agricultural science and technology innovation, and produce more scientific and comprehensive selection results.

### Management implications

This research yields dual managerial contributions. Primarily, it offers actionable insights for corporate decision-makers selecting digital technology collaborators during agricultural digitization initiatives. Concurrently, the investigation introduces a dynamic partnership framework grounded in field theory, specifically designed to optimize innovation alliances. The proposed model empowers DAST Innovation Consortium participants to effectively implement collaborative digital R&D strategies, particularly within the context of advancing sustainable agricultural modernization. The study’s applied value manifests in two dimensions:

This research develops a new methodology for partner selection in DAST innovation alliances’ collaborative digital innovation processes. Alliance participants frequently encounter challenges when choosing appropriate collaborators for scientific and technological advancement. The investigation presents an evolving partner selection framework that assists DAST alliance members in identifying optimal digital co-creation partners from extensive candidate pools, emphasizing strategic decision-making criteria and resource synergy. This study draws upon complementary multi-dimensional perspectives from relevant research, including technology adoption, socio-organizational drivers, and the role of agriculture in urban and planning-oriented sustainability frameworks, incorporating resource complementarity into the research framework^75,76^.The proposed approach utilizes grey correlation analysis combined with entropy weighting to calculate attribute significance, enhancing measurement precision. The methodology incorporates temporal dynamics into a multi-criteria optimization framework for weight allocation, resolving chronological weighting factors to effectively integrate multi-phase decision data while minimizing information ambiguity in aggregated temporal datasets. Evaluation mechanisms employing fuzzy logic systems integrated with vector projection techniques assess both existing alliance members and potential collaborators. Subsequently, a resource synergy evaluation matrix is introduced to identify optimal partnership configurations, enabling practical implementation of digital agricultural technology co-creation initiatives. The framework offers strategic recommendations for enhancing adaptive partner selection mechanisms and establishing sustainable collaborative networks within DAST innovation ecosystems.

### Limitations and future directions

In the upcoming studies, we will apply this method to practical problems, including the dynamic partner selections of I-U-R alliances, industrial innovation alliances, and other strategic alliances. We will also plan to expand this model to analyze dynamic multi-attribute decision-making problems with other types of fuzzy numbers such as triangular intuitionistic fuzzy numbers, interval intuitionistic fuzzy numbers, or generalized orthogonal fuzzy numbers. As the current era increasingly emphasizes AI-driven decision-making, integrating adaptive AI models with existing MCDM or fuzzy frameworks could significantly enhance the robustness, scalability, and real-time adaptability of decision support systems. While the current study establishes a dynamic MCDM-based evaluation method, the rapid evolution of Digital Agriculture necessitates intelligent adaptive mechanisms. In future, we should draw inspiration from recent advances, such as, real-time monitoring^77^, and smart region decision support systems^78^, use computer vision and deep Learning to monitor alliance partners’ operational compliance in real-time. In addition, we can use reinforcement learning algorithms to train on historical alliance performance data to dynamically adjust criteria weights, addressing the temporal volatility of agricultural innovation environments.

## Conclusion

Drawing upon the principles of intuitionistic fuzzy theory and field theory, this research constructs a dynamic decision framework incorporating dual-combination weighting approach integrated with temporal adjustments for DAST innovation alliance partner selection. An empirical case study involving China’s 5G Agricultural Digitalization Alliance partnership evaluation demonstrates the model’s practical application. Comparative assessment with conventional methodologies confirms the proposed approach’s validity and operational effectiveness in real-world decision scenarios. The investigation yields four key conclusions:

The integrated weighting approach successfully incorporates both decision-makers’ subjective preferences and objective data, leading to enhanced attribute weighting in the proposed model. By utilizing gray relational analysis combined with intuitionistic fuzzy entropy calculations for objective weighting across temporal phases, this methodology significantly enhances the precision and reliability of attribute weight determinations in multi-period decision scenarios.

This research incorporates decision-makers’ preferential weighting of temporal sequences and multi-phase data assimilation to develop a multi-objective optimization framework that determines the optimal temporal weighting vector. This approach enables the framework to systematically integrate heterogeneous data inputs across progressive stages, thereby reducing instability caused by temporal information convergence. The proposed methodology strategically addresses variance amplification resulting from multi-temporal sample aggregation through dynamic parameter calibration and phased information absorption mechanisms.

The orthogonal projection technique is employed to compute vertical distances for evaluating existing and potential DAST innovation alliance collaborators. This approach addresses a key limitation inherent in traditional TOPSIS methodologies by simultaneously evaluating proximity to both ideal and non-ideal reference points. The enhanced methodology identifies optimal solutions that demonstrate maximal alignment with positive benchmarks while maintaining maximal separation from negative benchmarks^73,74^. This dual-criteria assessment framework provides a more holistic evaluation of solution viability compared to conventional approaches, ensuring enhanced rationality in partner selection through comprehensive analysis of relative positioning within the solution space;

The developed approach systematically accounts for synergistic resource dynamics within DAST innovation ecosystems, establishing essential entry/exit criteria for collaborative entities while constructing an evolving partner selection framework grounded in field theory principles. This mechanism evaluates resource alignment potential between existing alliances and prospective collaborators to facilitate adaptive partner curation, enabling continuous optimization of innovation networks through merit-based evaluation processes that ensure optimal partnership configurations.

## Data Availability

The data supporting the findings of this study can be obtained upon request from the corresponding author. Please note that the data are not publicly accessible due to privacy and ethical considerations.

## Appendix

See Tables 8, 9, 10, 11, 12 and 13.

**Table 8:** Semantic Information in T1.

	Resource complementary level	Knowledge- sharing level	The cultural similarity	Risk-sharing ability	Cooperation compatibility ability
R1	Good	Moderate Good	Moderate Good	Good	Good
R2	Very Good	Good	Best	Good	Excellent
R3	Moderate Good	Very Good	Excellent	Moderate Good	Very Good
R4	Very Good	Moderate Bad	Moderate Good	Common	Good
R5	Moderate Bad	Common	Moderate Good	Very Good	Common
R6	Common	Common	Very Good	Moderate Bad	Common
R7	Common	Moderate Good	Excellent	Common	Very Good
R8	Moderate Bad	Very Good	Good	Common	Common
R9	Good	Common	Common	Very Good	Moderate Bad
R10	Common	Excellent	Moderate Bad	Good	Common
S1	Very Good	Good	Good	Moderate Good	Very Good
S2	Moderate Good	Common	Excellent	Common	Good
S3	Very Good	Common	Moderate Good	Very Good	Good
S4	Good	Good	Moderate Good	Very Good	Moderate Good
S5	Moderate Good	Excellent	Good	Common	Common

**Table 9:** Semantic Information in T2.

	Resource complementary level	Knowledge- sharing level	The cultural similarity	Risk-sharing ability	Cooperation compatibility ability
R1	Very Good	Good	Moderate Bad	Common	Good
R2	Common	Excellent	Good	Moderate Good	Good
R3	Very Good	Excellent	Common	Excellent	Good
R4	Common	Common	Very Good	Common	Moderate Bad
R5	Common	Common	Moderate Bad	Common	Very Good
R6	Good	Common	Excellent	Moderate Good	Good
R7	Moderate Bad	Very Good	Good	Good	Moderate Bad
R8	Excellent	Moderate Bad	Very Good	Moderate Good	Common
R9	Moderate Bad	Moderate Bad	Very Good	Common	Common
R10	Good	Good	Common	Very Good	Good
S1	Good	Moderate Good	Very Good	Moderate Good	Good
S2	Common	Good	Common	Very Good	Common
S3	Moderate Good	Very Good	Good	Moderate Good	Excellent
S4	Good	Moderate Good	Very Good	Good	Moderate Good
S5	Good	Common	Excellent	Good	Very Good

**Table 10:** Semantic Information in T3.

	Resource complementary level	Knowledge- sharing level	The cultural similarity	Risk-sharing ability	Cooperation compatibility ability
R1	Moderate Bad	Very Good	Common	Good	Common
R2	Moderate Bad	Best	Moderate Good	Moderate Good	Good
R3	Moderate Good	Moderate Good	Common	Good	Very Good
R4	Moderate Good	Common	Moderate Bad	Very Good	Moderate Good
R5	Good	Very Good	Moderate Good	Moderate Bad	Common
R6	Very Good	Common	Moderate Good	Very Good	Common
R7	Good	Very Good	Moderate Good	Very Good	Good
R8	Good	Moderate Good	Very Good	Good	Good
R9	Very Good	Good	Good	Moderate Good	Excellent
R10	Moderate Good	Very Good	Common	Good	Very Good
S1	Good	Moderate Good	Good	Moderate Good	Common
S2	Moderate Good	Moderate Good	Good	Very Good	Moderate Good
S3	Good	Very Good	Moderate Good	Common	Good
S4	Moderate Good	Good	Very Good	Good	Good
S5	Very Good	Moderate Good	Moderate Good	Good	Excellent

**Table 11:** The inter-rater reliability statistics results in T1 time period.

Dimension	Kendall’s W	χ^2^	Maximum deviation	Average deviation	Range of expert mean	P-value	ICC	Consistency level
Resource complementary	0.714	152.12	0.27	0.13	3.9–8.1	< 0.001	0.729	High consistency
Knowledge sharing	0.660	146.80	0.27	0.12	3.9–8.9	< 0.001	0.674	Moderate consistency
Cultural similarity	0.770	160.09	0.27	0.17	4.3–9.7	< 0.001	0.767	High consistency
Risk sharing	0.619	134.51	0.27	0.16	3.7–8.2	< 0.001	0.636	Moderate consistency
Cooperation compatibility	0.698	141.90	0.27	0.12	4.1–8.1	< 0.001	0.682	Moderate consistency

**Table 12:** The inter-rater reliability statistics results in T2 time period.

Dimension	Kendall’s W	χ^2^	Maximum deviation	Average deviation	Range of expert mean	P-value	ICC	Consistency level
Resource complementary	0.736	157.35	0.27	0.16	3.9–8.7	< 0.001	0.730	High consistency
Knowledge sharing	0.735	160.04	0.27	0.16	4.0–9.1	< 0.001	0.741	High consistency
Cultural similarity	0.756	157.54	0.27	0.10	3.9–9.2	< 0.001	0.792	High consistency
Risk sharing	0.580	125.32	0.27	0.13	4.9–9.2	< 0.001	0.621	Moderate consistency
Cooperation compatibility	0.703	151.56	0.27	0.11	3.7–9.0	< 0.001	0.725	High consistency

**Table 13:** The inter-rater reliability statistics results in T3 time period.

Dimension	Kendall’s W	χ^2^	Maximum deviation	Average deviation	Range of expert mean	P-value	ICC	Consistency level
Resource complementary	0.726	153.09	0.13	0.07	4.0–8.1	< 0.001	0.814	High consistency
Knowledge sharing	0.828	166.91	0.20	0.07	5.0–9.8	< 0.001	0.868	High consistency
Cultural similarity	0.695	150.74	0.13	0.08	3.9–7.9	< 0.001	0.759	High consistency
Risk sharing	0.738	159.03	0.20	0.07	3.9–8.2	< 0.001	0.810	High consistency
Cooperation compatibility	0.832	173.12	0.33	0.09	4.7–9.0	< 0.001	0.856	High consistency

## Abbreviations

DAST: Digital Agricultural Science and Technology
MCDA: Multi-criteria decision analysis
IoT: Internet of Things
5G: 5Th Generation
AST: Agricultural Science and Technology
I-U-R: Industry-university-research
TOPSIS: Technique for Order Preference by Similarity to an Ideal Solution
R&D: Research and Development
API: Application Programming Interface
NGOs: Non-Governmental Organizations
G20: Group of Twenty
MCDM: Multiple Criteria Decision Making
HFS: Hesitation fuzzy sets
DFT: Decision field theory
HFDFT: Hesitation fuzzy decision field theory
DFT-L: Dynamic Fuzzy TOPSIS with Linguistic variables
ILOR: Linear ordered ranking
DEMATEL-AEW-FVIOR: Decision-Making Trial and Evaluation Laboratory - Analytic Entropy Weight - Fuzzy VIKOR
LBWA: Level Based Weight Assessment
COPRAS: Complex Proportional Assessment
AHP-CoCoSo: Analytic Hierarchy Process - Combined Compromise Solution
SWARA: Step-Wise Weight Assessment Ratio Analysis
WASPAS: Weighted Aggregated Sum Product Assessment
SF: Spherical fuzzy
FFSs: Fermatean Fuzzy Sets
FF-TOPSIS: Fermatean Fuzzy TOPSIS
MCGDM: Multi-criteria group decision-making
MSME: Medium and Small-sized Micro and Small Enterprises
MADM: Multiple attribute decision-making
IFSs: Intuitionistic fuzzy sets
IFNs: Intuitionistic Fuzzy Numbers
DIFS: Dynamic intuitionistic fuzzy set
DIFWG: Dynamic intuitionistic fuzzy weighted
AI: Artificial Intelligence
ICC: Intraclass Correlation
